# Nationwide medical database study for postoperative nutritional management in patients undergoing gastroenterological cancer surgery

**DOI:** 10.1002/ags3.12892

**Published:** 2024-11-27

**Authors:** Yoshikuni Kawaguchi, Kenta Murotani, Nahoki Hayashi, Satoru Kamoshita

**Affiliations:** ^1^ Hepato‐Biliary‐Pancreatic Surgery Division, Department of Surgery, Graduate School of Medicine The University of Tokyo Tokyo Japan; ^2^ School of Medical Technology Kurume University Fukuoka Japan; ^3^ Biostatistics Center Kurume University Fukuoka Japan; ^4^ Medical Affairs Department Research and Development Center, Otsuka Pharmaceutical Factory Tokyo Japan

**Keywords:** early oral intake, fasting, gastroenterological cancer surgery, postoperative nutritional management, real‐world data

## Abstract

**Aim:**

The study aimed to clarify how nutrition was managed in patients based on surgical site during the first 7 d after gastroenterological cancer surgery.

**Methods:**

A Japanese medical claims database was used to identify patients who had gastroenterological cancer surgery from 2011 to 2022. Patients were divided into groups based on the surgical sites, and postoperative feeding routes and timing of initiation of oral intake for groups were assessed. For the subset of patients fasting postoperatively for ≥7 d, the proportion of patients prescribed target doses of energy (20 kcal/kg) and amino acids (0.8 g/kg) on postoperative d 7 were assessed.

**Results:**

Surgical sites of 360 296 study patients were: esophagus, 14 784; stomach, 103 339; colon/rectum, 194 049; liver, 19 277; gallbladder/bile duct, 8279; pancreas, 20 568. The median postoperative day of oral intake initiation was: esophagus, seven; stomach and pancreas, four; colon/rectum and gallbladder/bile duct, three; liver, two. The proportions of fasting patients prescribed target doses of energy and amino acids on postoperative d 7 were: esophagus, 42.6% and 34.4%; stomach, 21.8% and 28.0%; colon/rectum, 20.9% and 29.1%; liver, 21.2% and 22.5%; gallbladder/bile duct, 31.0% and 33.4%; pancreas, 28.2% and 37.8%, respectively.

**Conclusion:**

Oral intake after gastroenterological cancer surgery was started earliest in patients undergoing liver surgery and latest in patients undergoing esophageal surgery. Target parenteral energy and amino acid doses were prescribed to less than half of fasting patients. Education is needed to promote early initiation of oral intake and the use of guidelines‐based parenteral nutrition dosing in patients after gastroenterological cancer surgery.

## Graphical abstract

The study aim was to clarify how nutrition was managed in patients based on surgical site during the first 7 d after gastrointestinal cancer surgery. As results, oral intake after gastrointestinal cancer surgery started earliest in patients having liver surgery and latest in patients having esophageal surgery. Substantially less than half of fasting patients were prescribed target parenteral energy and amino acid doses.

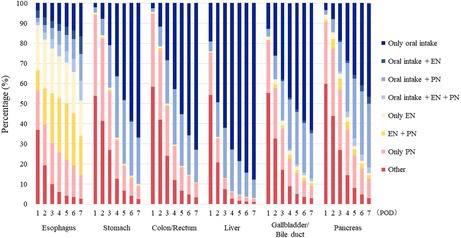

## 1 INTRODUCTION

Gastrointestinal (GI) cancers account for one‐fourth of all cancers and one‐third of all cancer‐related deaths in the world.^1^ Globally, the estimated lifetime incidence risk of GI cancer is 8.2% and the risk of GI cancer‐related death is 6.2% (ie, 1 in 12 persons will develop and 1 in 16 will die of GI cancer).^2^ The primary treatment option for cancer is surgery, and the number of patients having surgery for GI cancer has been increasing over time in Japan.^3^ After such surgery, GI function is impaired and food intake is often inadequate, both of which lead to a high risk of malnutrition.^4^, ^5^ Malnutrition in patients undergoing GI cancer surgery is associated with a decrease in the effectiveness of treatment, an increase in complications, and a worse prognosis.^5^ Thus, nutritional management after GI cancer surgery has an important role to play in preventing malnutrition, and is expected to decrease postoperative complications and enhance recovery after surgery.

Enhanced recovery after surgery (ERAS) programs are an evidence‐based approach of surgical care aimed at minimizing the stress of surgery and supporting quick recovery and are used increasingly throughout the world.^6^ A central tenet of these programs is the initiation of early oral intake. Similarly, early oral intake or enteral nutrition (EN) are recommended in the general nutritional guidelines of various countries. The recommended energy and protein intake for postsurgical patients is 20–30 kcal/kg/day and 0.8–1.5 g/kg/day, respectively.^7^, ^8^, ^9^

However, in terms of early oral intake or EN and the recommended intake of energy and protein, a “knowledge‐to‐action gap” seems to exist in the field of clinical nutrition.^10^ A survey using a medical claims database reported that 16.7% of patients on dialysis undergoing GI surgery received only parenteral nutrition (PN), without any oral intake or EN, even by the seventh day after surgery. In that survey, the median (interquartile range [IQR]) prescribed doses of parenteral energy and amino acids on the seventh day after surgery were 16.1 (5.8–24.6) kcal/kg/d and 0.32 (0.00–0.55) g/kg/day, respectively, both far below what guidelines recommend.^11^ The survey included only patients on dialysis and it grouped all patients together regardless of the site within the GI tract of their surgery. To our knowledge, no study investigated the nutritional management by surgical site in patients after gastroenterological cancer surgery.

As the surgical stress on patients differs by the part of the GI tract,^2^ we hypothesized that postoperative nutritional management would differ by surgical site. Accordingly, we conducted a study using a nationwide database on the nutritional management of patients after gastroenterological cancer surgery, and we grouped patients by the sites of their surgeries within the GI tract (ie, esophagus, stomach, colon/rectum, liver, gallbladder/bile duct, or pancreas). This study aimed to clarify how nutrition was managed in patient groups based on surgical site during the first 7 d after gastroenterological cancer surgery. We focused on postoperative feeding routes, timing of initiation of oral intake, risk factors for prolonged postoperative fasting, and prescribed parenteral doses of energy, amino acids, and lipid. The goal was to provide data about the current state of postsurgical nutritional management in patients undergoing gastroenterological cancer surgery as a means to promote improved evidence‐based nutritional management for this patient group.

## 2 METHODS

### 2.1 Study design and data source

This was a cohort study using a nationwide medical claims database (Medical Data Vision; Tokyo, Japan). The database covered ~ 42 million patients in 475 Japanese hospitals. These institutions represented ~27% of the acute care hospitals operating in Japan as of December 2022. The information contained in the database was as follows: dates of hospital admission and discharge, patient characteristics at hospital admission, medical treatments during hospitalization, and clinical outcomes at the time of discharge. For our study, diagnoses were identified using the International Classification of Diseases, 10th Revision (ICD‐10) codes. Medical treatments received during hospitalization were classified using Japan‐specific codes that appeared in medical claims.

### 2.2 Study patients

The study included adult patients who underwent gastroenterological cancer surgery under general anesthesia between January 2011 and December 2022, and patients were identified from the database whose surgical site location was consistent with their type of gastroenterological cancer, based on Japan‐specific medical claims codes (Table S1). Patients excluded from the study were those whose height or body weight data were missing or suspected to be the result of an input error (ie, height <100 or ≥200 cm, body weight <10 or ≥200 kg), who died or were discharged within postsurgical 7 d, or who underwent multisite surgery. The study patients were divided into the following six groups based on the surgical sites classified by the Japan‐specific codes within medical claims: esophagus, stomach, colon/rectum, liver, gallbladder/bile duct, and pancreas.

### 2.3 Extracted data

#### 2.3.1 Patient characteristics

Patient characteristics at the time of hospital admission were extracted from the database, including age, sex, height, body weight, beds in admission hospital, admission type, ICD‐10 disease diagnosis, activities of daily living (using the Barthel Index^12^), smoking history, and cancer stage (using the Tumor‐Node‐Metastasis [TNM] staging system^13^). Age was categorized as <60, 60–69, 70–79, 80–89, or ≥90 y. Body mass index (BMI) was calculated using height and body weight and categorized based on the World Health Organization (WHO) classification^14^ as <16, ≥16–<18.5, ≥18.5–<22.5, ≥22.5–<25, ≥25–<30, or ≥30. The number of beds in admission hospital was extracted as the categories of <200, 200–500, or ≥500 beds, and admission type was extracted as the categories of emergency or elective. The Charlson Comorbidity Index^15^, ^16^ was calculated using ICD‐10 disease diagnoses and categorized as 0–1, 2–3, 4–5, or ≥6. The Barthel Index expressed as the range from 0 (requiring full assistance) to 100 (requiring no assistance) was categorized as 0, 5–40, 45–60, 65–95, or 100. “Low BMI” was defined as BMI <18.5 in the patients <70 y old and BMI <20 in those ≥70 y old on the basis of the Asian Global Leadership Initiative on Malnutrition criteria.^17^ The level of food intake independence was categorized as 10 (requiring no assistance), 5 (requiring partial assistance), or 0 (requiring full assistance), based on the Barthel Index Feeding score.

#### 2.3.2 Preoperative medical treatments

Information about preoperative treatments was extracted from the database based on Japan‐specific medical claims codes (Table S1). These treatments included oral management (support for oral intake functions, including swallowing and chewing),^18^ EN (tube feeding), and PN (intravenous solutions containing amino acids and/or lipid), all from the day of hospital admission through the day before surgery. In addition, these included preoperative cancer treatments (ie, chemotherapy and/or radiation therapy) received from 60 d before surgery through the day before surgery.

#### 2.3.3 Day of surgery data

Data about surgical methods, infusions, and intensive care unit (ICU) admission on the surgery day were extracted from the database based on Japan‐specific medical claims codes (Table S1). These included surgical methods (laparoscopic or open surgery), intravenous fluid infusions (crystalloid, colloid [dextrans and/or hydroxyethyl starches], and/or albumin), transfusions (red blood cells, platelets, and/or fresh‐frozen plasma), and ICU admission. Crystalloid fluid infusions were categorized based on prescribed volumes as ≤5000, >5000–≤10 000, or >10 000 mL. Colloid fluid, albumin, and transfusions were categorized based on prescribed volumes as 0, >0–≤500, or >500 mL.

### 2.4 Evaluation of nutritional management

#### 2.4.1 Postoperative feeding routes

Information about daily feeding routes and prescribed nutrition doses was extracted from the database based on Japan‐specific medical claims codes that appeared in medical claims (Table S1). Feeding routes were expressed as either oral intake (defined as meals served), EN, PN, or a combination of these. Data not fitting into one of those categories (eg, prescribed fluids containing only carbohydrate and/or electrolytes) were expressed as “Other.”

Postoperative day (POD) 1 was defined as the next day after surgery, and the proportions of patients receiving each feeding route (ie, oral intake, EN, PN, combination, or other) in each surgical site group were calculated for each day from POD1 through POD7. Also, the proportions of patients in each group receiving oral intake, EN, PN, or supplemental parenteral nutrition (SPN; defined as PN prescribed on the same day as oral intake and/or EN) were calculated for the periods of POD1–3, 1–5, and 1–7. Finally, the postoperative day of initiation of oral intake was determined for each patient and then the proportions of patients starting oral intake on each day (ie, POD1 through >7) were calculated for each surgical site group.

#### 2.4.2 Risk factors for postoperative fasting of 7 d or longer

First, all patients were divided into two groups: those who received oral intake or EN by POD7, and those who did not receive oral intake or EN by POD7 (ie, postoperative fasting ≥7 d). Then the risk for postoperative fasting ≥7 d was estimated for different patient characteristics, preoperative medical treatments, and surgery day data using multivariable logistic regression analysis.

#### 2.4.3 Postoperative parenteral nutrition doses and target dose attainment

For patients who were fasting ≥7 d after surgery, the prescribed doses based on the ideal body weight of parenteral energy (kcal/kg), amino acids (g/kg), and lipid (g/kg) were calculated for the period of POD1 to POD7. In addition, the proportions of patients in each surgical site group who were prescribed guideline‐recommended target parenteral energy (≥20 kcal/kg) and amino acid (≥0.8 g/kg) doses^7^ were calculated for POD7.

### 2.5 Statistical analysis

Continuous variables were described using medians and interquartile ranges (IQR; first quartile [Q1], third quartile [Q3]) or means and standard deviations (SD). Categorical variables were reported using frequencies and proportions. Missing data were treated without replacement. For the estimation of risk factors for postoperative fasting ≥7 d, multivariable logistic regression analysis was performed using fasting as the objective variable and patient characteristics, preoperative medical treatments, and day of surgery data as the explanatory variables. The confidence coefficient for the confidence interval calculation was set at 95%. All statistical analyses were performed using SAS, v. 9.4 (SAS Institute, Cary, NC, USA). An independent third party, A2 Healthcare Corporation (Tokyo, Japan), conducted data management and statistical analysis.

## 3 RESULTS

### 3.1 Patient characteristics, preoperative medical treatments, day of surgery data

**FIGURE 1:**
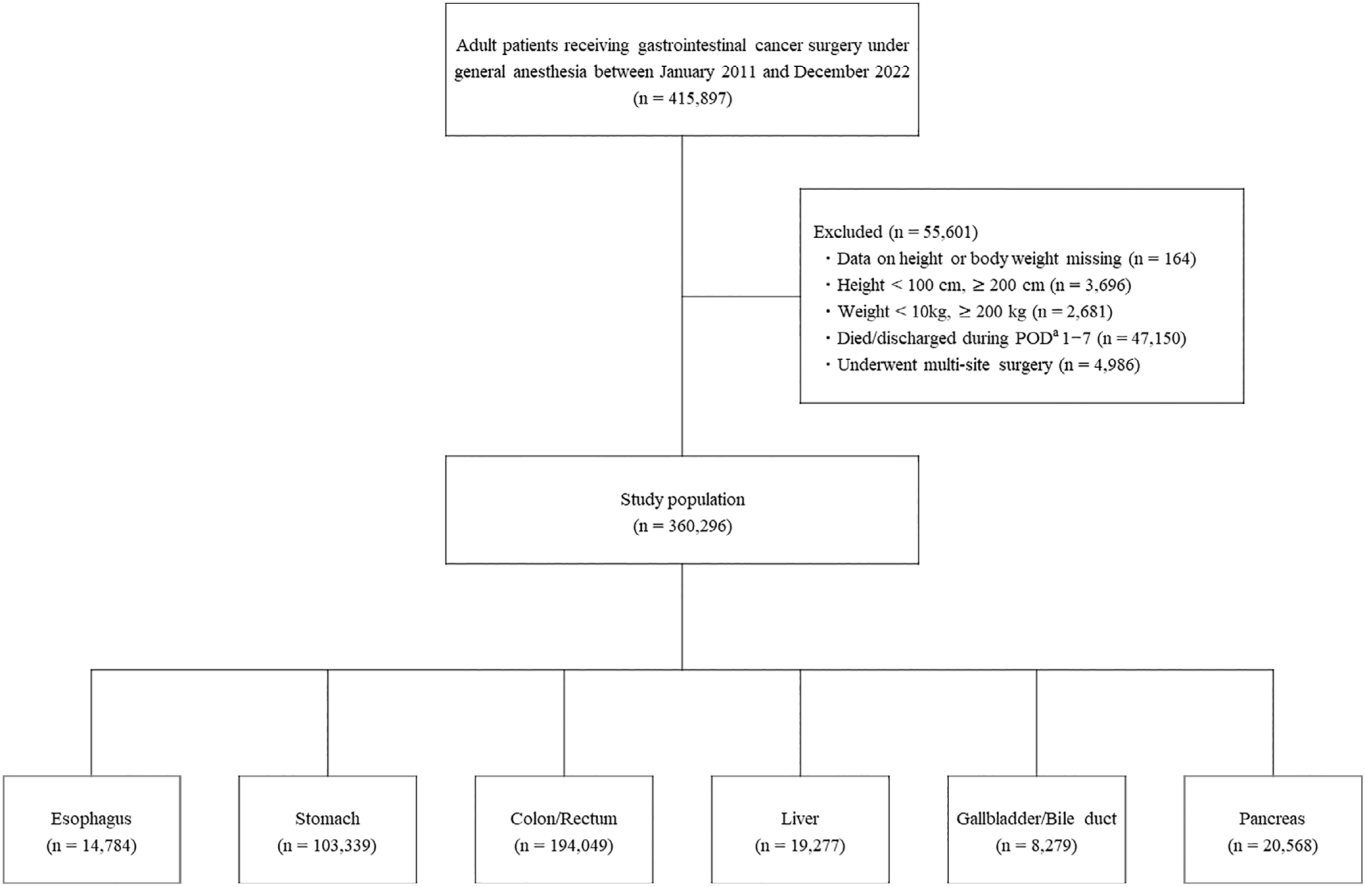
Study flow chart and disposition by surgical site of 360 296 adult patients in Japan who underwent gastroenterological cancer surgery from 2011 to 2022. ^a^Postoperative day (POD) 1 defined as the next day after surgery.

Of 415 897 patients who underwent gastroenterological cancer surgery under general anesthesia between January 2011 and December 2022, 360 296 patients were included in the study. Of those, the numbers of patients undergoing surgery of the esophagus, stomach, colon/rectum, liver, gallbladder/bile duct, and pancreas were 14 784 (4.1%), 103 339 (28.7%), 194 049 (53.9%), 19 277 (5.4%), 8279 (2.3%), and 20 568 (5.7%), respectively (Figure 1). Patient characteristics and preoperative medical treatments received are shown in Table 1, and day of surgery data (ie, surgical methods, infusions, and ICU admission) are shown in Table 2.

**TABLE 1:** Demographic characteristics and preoperative medical treatments of 360 296 adult patients in Japan who underwent gastroenterological cancer surgery from 2011 to 2022, by surgical site.

Characteristics	Variable categories	Esophagus	Stomach	Colon/Rectum	Liver	Gallbladder/Bile duct	Pancreas
		*N* = 14 784	*N* = 103 339	*N* = 194 049	*N* = 19 277	*N* = 8279	*N* = 20 568
		*n* (%)	*n* (%)	*n* (%)	*n* (%)	*n* (%)	*n* (%)
Age, y	18–59	2540 (17.2)	12 874 (12.5)	29 799 (15.4)	1994 (10.3)	654 (7.9)	2744 (13.3)
	60–69	5255 (35.5)	26 916 (26.0)	49 614 (25.6)	5122 (26.6)	1823 (22.0)	5608 (27.3)
	70–79	5782 (39.1)	40 186 (38.9)	68 988 (35.6)	8827 (45.8)	3744 (45.2)	9176 (44.6)
	80–89	1194 (8.1)	21 915 (21.2)	40 849 (21.1)	3270 (17.0)	1937 (23.4)	3005 (14.6)
	≥90	13 (0.1)	1448 (1.4)	4799 (2.5)	64 (0.3)	121 (1.5)	35 (0.2)
Sex	Male	12 087 (81.8)	70 905 (68.6)	112 072 (57.8)	14 344 (74.4)	4873 (58.9)	11 498 (55.9)
	Female	2697 (18.2)	32 434 (31.4)	81 977 (42.2)	4933 (25.6)	3406 (41.1)	9070 (44.1)
BMI	<16	548 (3.7)	2060 (2.0)	4394 (2.3)	140 (0.7)	133 (1.6)	426 (2.1)
	≥16, <18.5	2122 (14.4)	10 047 (9.7)	19 699 (10.2)	1002 (5.2)	701 (8.5)	2300 (11.2)
	≥18.5, <22.5	6663 (45.1)	41 900 (40.5)	76 295 (39.3)	6243 (32.4)	3336 (40.3)	9251 (45.0)
	≥22.5, <25	3354 (22.7)	27 196 (26.3)	48 860 (25.2)	5487 (28.5)	2228 (26.9)	4911 (23.9)
	≥25, <30	1934 (13.1)	19 663 (19.0)	37 964 (19.6)	5334 (27.7)	1639 (19.8)	3232 (15.7)
	≥30	163 (1.1)	2473 (2.4)	6837 (3.5)	1071 (5.6)	242 (2.9)	448 (2.2)
Beds in admission hospital	<200	324 (2.2)	5690 (5.5)	12 221 (6.3)	755 (3.9)	338 (4.1)	386 (1.9)
	≥200, <500	5705 (38.6)	55 566 (53.8)	108 684 (56.0)	7855 (40.7)	4186 (50.6)	9139 (44.4)
	≥500	8755 (59.2)	42 083 (40.7)	73 144 (37.7)	10 667 (55.3)	3755 (45.4)	11 043 (53.7)
Admission type	Elective	14 279 (96.6)	94 497 (91.4)	160 968 (83.0)	18 853 (97.8)	6866 (82.9)	18 781 (91.3)
	Emergency	191 (1.3)	5467 (5.3)	20 873 (10.8)	234 (1.2)	856 (10.3)	875 (4.3)
	NA	314 (2.1)	3375 (3.3)	12 208 (6.3)	190 (1.0)	557 (6.7)	912 (4.4)
Charlson Comorbidity Index	0–1	535 (3.6)	3118 (3.0)	8325 (4.3)	771 (4.0)	945 (11.4)	1543 (7.5)
	2–3	12 090 (81.8)	84 277 (81.6)	148 756 (76.7)	7629 (39.6)	5784 (69.9)	15 806 (76.8)
	4–5	1233 (8.3)	9492 (9.2)	17 039 (8.8)	9353 (48.5)	1055 (12.7)	2247 (10.9)
	≥6	926 (6.3)	6452 (6.2)	19 929 (10.3)	1524 (7.9)	495 (6.0)	972 (4.7)
Barthel Index	100	13 986 (94.6)	92 050 (89.1)	162 250 (83.6)	17 806 (92.4)	7114 (85.9)	19 140 (93.1)
	65–95	413 (2.8)	4904 (4.7)	11 586 (6.0)	679 (3.5)	444 (5.4)	754 (3.7)
	45–60	91 (0.6)	1710 (1.7)	4974 (2.6)	194 (1.0)	169 (2.0)	191 (0.9)
	5–40	41 (0.3)	1220 (1.2)	4228 (2.2)	94 (0.5)	158 (1.9)	94 (0.5)
	0	44 (0.3)	1076 (1.0)	4625 (2.4)	91 (0.5)	171 (2.1)	78 (0.4)
	NA	209 (1.4)	2379 (2.3)	6386 (3.3)	413 (2.1)	223 (2.7)	311 (1.5)
Smoking history	Yes	9536 (64.5)	45 859 (44.4)	72 129 (37.2)	9280 (48.1)	2815 (34.0)	7914 (38.5)
	No	3857 (26.1)	48 773 (47.2)	106 060 (54.7)	8312 (43.1)	4755 (57.4)	11 095 (53.9)
	NA	1391 (9.4)	8707 (8.4)	15 860 (8.2)	1685 (8.7)	709 (8.6)	1559 (7.6)
Low BMI^a^	Yes	3706 (25.1)	19 923 (19.3)	38 445 (19.8)	2191 (11.4)	1553 (18.8)	4486 (21.8)
	No	11 078 (74.9)	83 416 (80.7)	155 604 (80.2)	17 086 (88.6)	6726 (81.2)	16 082 (78.2)
TNM cancer classification	I	1825 (12.3)	24 392 (23.6)	40 091 (20.7)	3270 (17.0)	1165 (14.1)	3798 (18.5)
	II	1911 (12.9)	9389 (9.1)	50 355 (25.9)	6657 (34.5)	2485 (30.0)	7471 (36.3)
	III	2653 (17.9)	9901 (9.6)	58 185 (30.0)	2915 (15.1)	1780 (21.5)	2833 (13.8)
	IV	554 (3.7)	3799 (3.7)	21 756 (11.2)	975 (5.1)	692 (8.4)	2199 (10.7)
	NA	7841 (53.0)	55 858 (54.1)	23 662 (12.2)	5460 (28.3)	2157 (26.1)	4267 (20.7)
Level of food intake independence	Required no assistance	14 430 (97.6)	98 791 (95.6)	178 785 (92.1)	18 694 (97.0)	7740 (93.5)	20 069 (97.6)
	Required partial assistance	128 (0.9)	2375 (2.3)	7112 (3.7)	243 (1.3)	224 (2.7)	218 (1.1)
	Required full assistance	110 (0.7)	1401 (1.4)	5788 (3.0)	113 (0.6)	212 (2.6)	118 (0.6)
	NA	116 (0.8)	772 (0.7)	2364 (1.2)	227 (1.2)	103 (1.2)	163 (0.8)
Preoperative oral management^b^, ^c^	Yes	6614 (44.7)	30 591 (29.6)	51 137 (26.4)	5054 (26.2)	1869 (22.6)	6589 (32.0)
Preoperative artificial nutrition^b^	Enteral nutrition^d^	873 (5.9)	1625 (1.6)	4731 (2.4)	123 (0.6)	106 (1.3)	213 (1.0)
	Parenteral nutrition^e^	2734 (18.5)	14 733 (14.3)	63 834 (32.9)	941 (4.9)	1156 (14.0)	2190 (10.6)
Preoperative cancer treatment^f^	Chemotherapy	6444 (43.6)	4561 (4.4)	7941 (4.1)	1055 (5.5)	248 (3.0)	4712 (22.9)
	Radiation therapy	513 (3.5)	681 (0.7)	2654 (1.4)	65 (0.3)	25 (0.3)	497 (2.4)

Abbreviations: BMI, body mass index; NA, not available.

^a^ Low BMI defined as BMI <18.5 in the patients <70 y old and BMI <20 in those ≥70 y old.

^**b**^ Received from the day of hospital admission through the day before surgery.

^c^ Support for oral intake functions, including swallowing and chewing.

^d^ Enteral nutrition defined as tube feedings prescribed.

^e^ Parenteral nutrition defined as intravenous solutions containing amino acids and/or lipid prescribed.

^**f**^ Received from 60 d before surgery through the day before surgery.

**TABLE 2:** Characteristics of treatments on day of surgery of 360 296 adult patients in Japan who underwent gastroenterological cancer surgery from 2011 to 2022, by surgical site.

Treatment characteristics	*Variable categories*	Esophagus	Stomach	Colon/Rectum	Liver	Gallbladder/Bile duct	Pancreas
		*N* = 14 784	*N* = 103 339	*N* = 194 049	*N* = 19 277	*N* = 8279	*N* = 20 568
		*n* (%)	*n* (%)	*n* (%)	*n* (%)	*n* (%)	*n* (%)
Surgical methods	Laparoscopic	9574 (64.8)	47 682 (46.1)	124 835 (64.3)	4155 (21.6)	518 (6.3)	1461 (7.1)
	Open	5210 (35.2)	55 657 (53.9)	69 214 (35.7)	15 122 (78.4)	7761 (93.7)	19 107 (92.9)
Prescribed infusions on day of surgery	Crystalloid fluid (mL)						
	≤ 5000	2597 (17.6)	26 478 (25.6)	57 597 (29.7)	2836 (14.7)	1182 (14.3)	1270 (6.2)
	>5000, ≤10 000	7805 (52.8)	65 219 (63.1)	113 696 (58.6)	11 998 (62.2)	4004 (48.4)	9498 (46.2)
	>10 000	4382 (29.6)	11 642 (11.3)	22 756 (11.7)	4443 (23.0)	3093 (37.4)	9800 (47.6)
	Colloid fluid^a^ (mL)						
	0	4738 (32.0)	50 244 (48.6)	107 595 (55.4)	7283 (37.8)	2795 (33.8)	5075 (24.7)
	>0, ≤500	3604 (24.4)	32 043 (31.0)	55 145 (28.4)	5627 (29.2)	2165 (26.2)	5550 (27.0)
	>500	6442 (43.6)	21 052 (20.4)	31 309 (16.1)	6367 (33.0)	3319 (40.1)	9943 (48.3)
	Albumin (mL)						
	0	10 653 (72.1)	96 332 (93.2)	182 242 (93.9)	14 979 (77.7)	6232 (75.3)	14 498 (70.5)
	>0, ≤ 500	3045 (20.6)	5855 (5.7)	9471 (4.9)	3139 (16.3)	1420 (17.2)	4273 (20.8)
	>500	1086 (7.3)	1152 (1.1)	2336 (1.2)	1159 (6.0)	627 (7.6)	1797 (8.7)
	Transfusion^b^ (mL)						
	0	12 582 (85.1)	91 677 (88.7)	175 437 (90.4)	14 571 (75.6)	6360 (76.8)	16 051 (78.0)
	>0, ≤ 500	899 (6.1)	4971 (4.8)	8280 (4.3)	1165 (6.0)	508 (6.1)	1349 (6.6)
	>500	1303 (8.8)	6691 (6.5)	10 332 (5.3)	3541 (18.4)	1411 (17.0)	3168 (15.4)
ICU admission on day of surgery	Yes	10 678 (72.2)	37 830 (36.6)	71 658 (36.9)	11 702 (60.7)	4504 (54.4)	13 569 (66.0)

Abbreviation: ICU, intensive care unit.

^a^ Prescribed dextrans and/or hydroxyethyl starches.

^b^ Prescribed blood cells, platelets, and/or fresh‐frozen plasma.

### 3.2 Postoperative feeding routes

**FIGURE 2:**
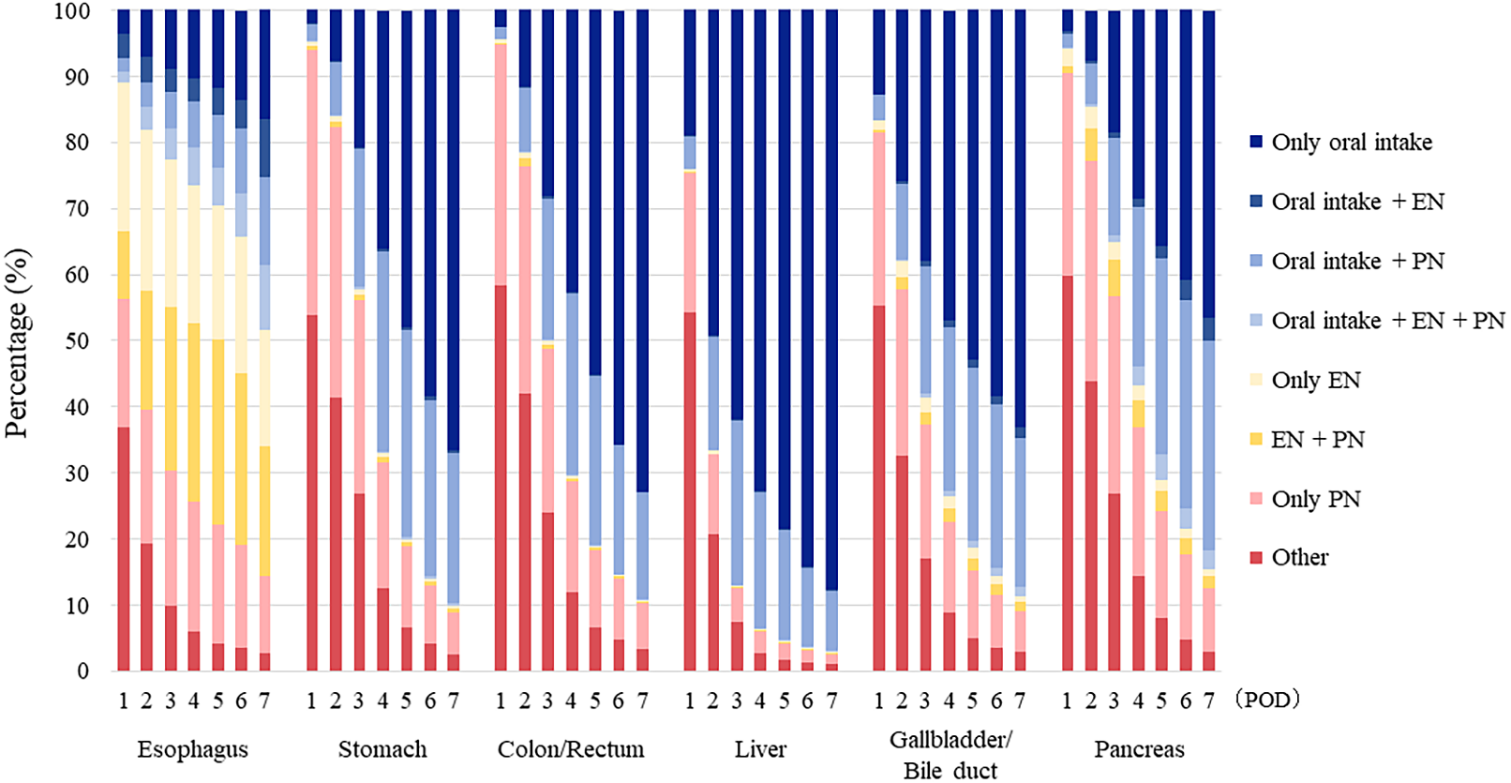
Feeding routes^a^ during postoperative d^b^ 1–7, received by 360 296 adult patients in Japan who underwent gastroenterological cancer surgery from 2011 to 2022, by surgical site.^c a^Oral intake defined as meals served; enteral nutrition (EN) defined as tube feedings prescribed; parenteral nutrition (PN) defined as intravenous solutions containing amino acids and/or lipid prescribed; and other defined as intravenous solutions containing only glucose and electrolytes prescribed. ^b^Postoperative day (POD) 1 defined as the next day after surgery. ^c^Groups (with number of patients) based on surgical sites: Esophagus (*n* = 14 784); stomach (*n* = 103 339); colon/rectum (*n* = 194 049); liver (*n* = 19 277); gallbladder/bile duct (*n* = 8279); and pancreas (*n* = 20 568).

Feeding routes (ie, oral intake, EN, PN, combination, or other) received by patients on each day of POD1 through 7 are shown in Figure 2 and Table S2. The proportions of patients receiving only oral intake, only EN, and only PN on POD3, by the site of surgery, were: esophagus, 8.9%, 22.4%, and 20.5%; stomach, 20.8%, 0.7%, and 29.4%; colon/rectum, 28.4%, 0.5%, and 24.9%; liver, 61.9%, 0.2%, and 5.1%; gallbladder/bile duct, 38.0%, 2.3%, and 20.1%; and pancreas, 18.4%, 2.8%, and 29.9%; all respectively. The difference of the feeding routes during postoperative d 1–7 by the surgical approach (ie, laparoscopic surgery vs open surgery) is shown in Figure S1. The proportion of patients who initiated oral intake on POD3 was higher in the patients who underwent laparoscopic surgery than in those who underwent open surgery for every surgical site (esophagus, 12.0% vs 3.3%; stomach, 27.0% vs 15.5%; colon/rectum, 32.1% vs 21.6%; liver, 75.8% vs 58.1%; gallbladder/bile duct, 71.8% vs 35.7%; pancreas, 33.5% vs 17.3%).

**FIGURE 3:**
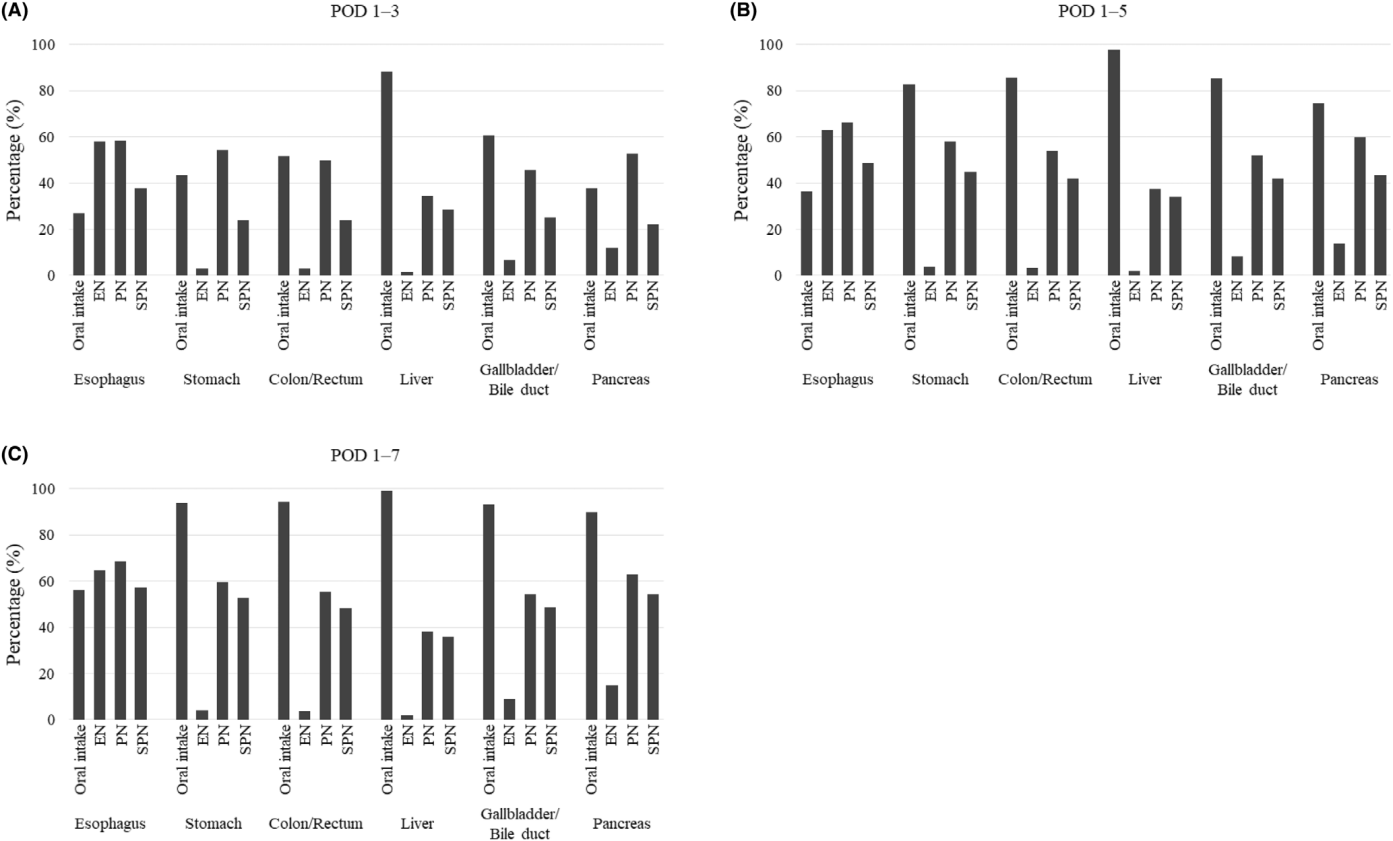
Feeding routes^a^ during postoperative d^b^ (A) 1–3, (B) 1–5, and (C) 1–7, received by 360 296 adult patients in Japan who underwent gastroenterological cancer surgery from 2011 to 2022, by surgical site.^c a^Oral intake defined as meals served; enteral nutrition (EN) defined as tube feedings prescribed; parenteral nutrition (PN) defined as intravenous solutions containing amino acids and/or lipid prescribed; and supplemental parenteral nutrition (SPN) defined as PN prescribed on same day as oral intake and/or EN. ^b^Postoperative day (POD) 1 defined as the next day after surgery. ^c^Groups (with number of patients) based on surgical sites: Esophagus (*n* = 14 784); stomach (*n* = 103 339); colon/rectum (*n* = 194 049); liver (*n* = 19 277); gallbladder/bile duct (*n* = 8279); and pancreas (*n* = 20 568).

Feeding routes (ie, oral intake, EN, PN, and SPN) received by patients for the periods POD1–3, POD1–5, and POD1–7 are shown in Figure 3 and Table S3. The proportions of patients receiving oral intake, EN, PN, and SPN, for the period POD1–3, by surgical site, were: esophagus, 27.0%, 58.0%, 58.4%, and 37.7%; stomach, 43.5%, 2.8%, 54.3%, and 24.1%; colon/rectum, 51.7%, 2.9%, 49.9%, and 24.1%; liver, 88.1%, 1.4%, 34.5%, and 28.4%; gallbladder/bile duct, 60.7%, 6.6%, 45.7%, and 25.2%, and pancreas, 37.6%, 11.9%, 52.9%, and 22.2%; all respectively.

**FIGURE 4:**
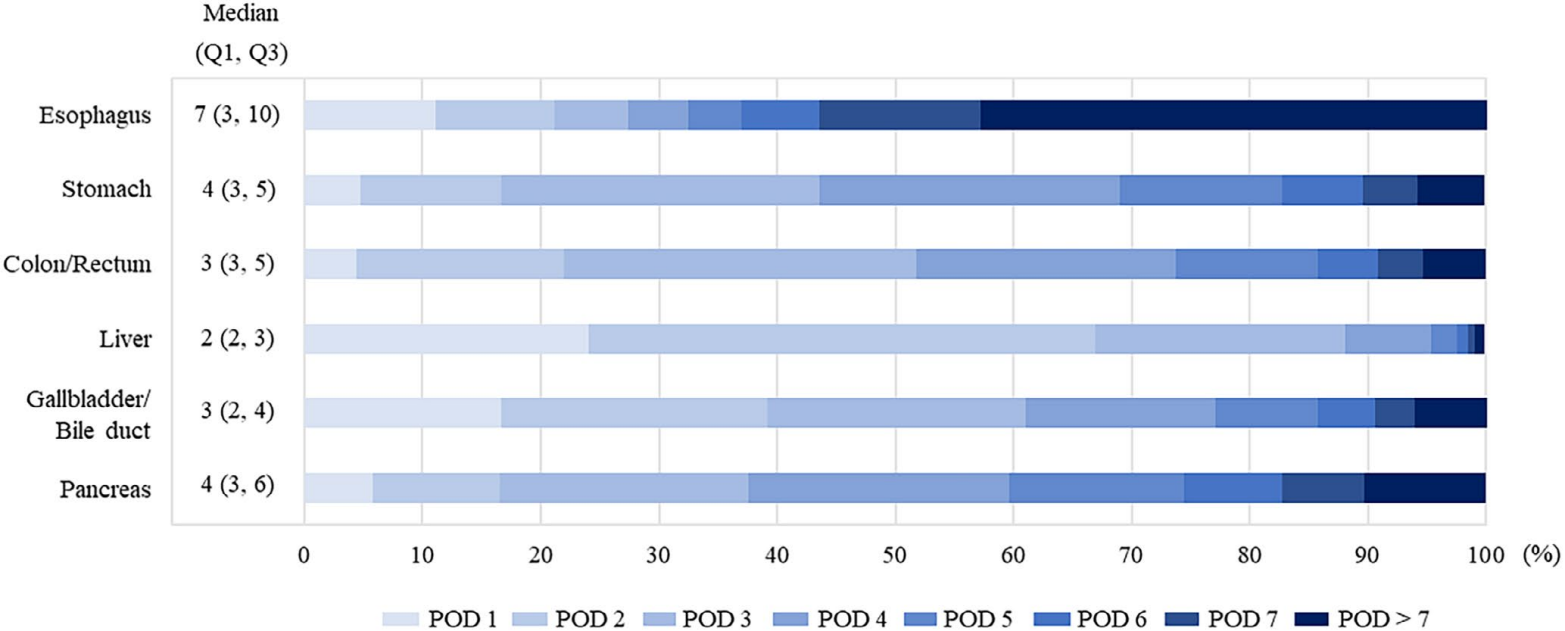
Postoperative day^a^ of initiation of oral intake among 359 138 adult patients in Japan who underwent gastroenterological cancer surgery and initiated oral intake during the hospitalized period from 2011 to 2022, by surgical site.^b^ Median (quartile 1 [Q1], quartile [Q3]) day of initiation of oral intake within each patient group, and distribution within patient groups of days of initiation of oral intake, both by surgical site. Postoperative day of initiation of oral intake was recorded as ranging from postoperative day (POD) 1 to POD >7. ^a^Postoperative day (POD) 1 defined as the next day after surgery. ^b^Groups (with numbers of patients) based on surgical sites: Esophagus (*n* = 14 537); stomach (*n* = 103 052); colon/rectum (*n* = 193 532); liver (*n* = 19 257); gallbladder/bile duct (*n* = 8229); and pancreas (*n* = 20 531).

The day that oral intake was initiated postoperatively is shown in Figure 4 and Table S4. The median (Q1, Q3) day that oral intake was started, by surgical site, was: esophagus, POD7 (3, 10); stomach, POD4 (3, 5); colon/rectum, POD3 (3, 5); liver, POD2 (2, 3); gallbladder/bile duct, POD3 (2, 4); and pancreas, POD4 (3, 6). The day of postoperative oral intake initiation by stratifying the presence or absence of postoperative complications is shown in Figure S2. The proportion of patients who initiated oral intake after POD7 was higher in the patients who developed postoperative complications than in those who did not for every surgical site (esophagus, 50.9% vs 39.5%; stomach, 16.0% vs 4.1%; colon/rectum, 14.5% vs 4.0%; liver, 2.8% vs 0.6%; gallbladder/bile duct, 10.5% vs 5.3%; pancreas, 15.0% vs 9.5%).

### 3.3 Risk factors for postoperative fasting of 7 d or longer

Based on multivariable logistic regression analysis, the significant independent risk factors for patients experiencing fasting ≥7 d after surgery were: age 80–89 y; male sex; BMI ≥22.5; <200 beds in admission hospital; emergency admission; Barthel Index <100; no preoperative oral management; preoperative PN; no preoperative chemotherapy; open surgery; crystalloid fluids >5000 mL on the surgery day; any colloids, albumins, or transfusions; and ICU admission on the surgery day (Table S5).

### 3.4 Postoperative parenteral nutrition doses and target dose attainment

**FIGURE 5:**
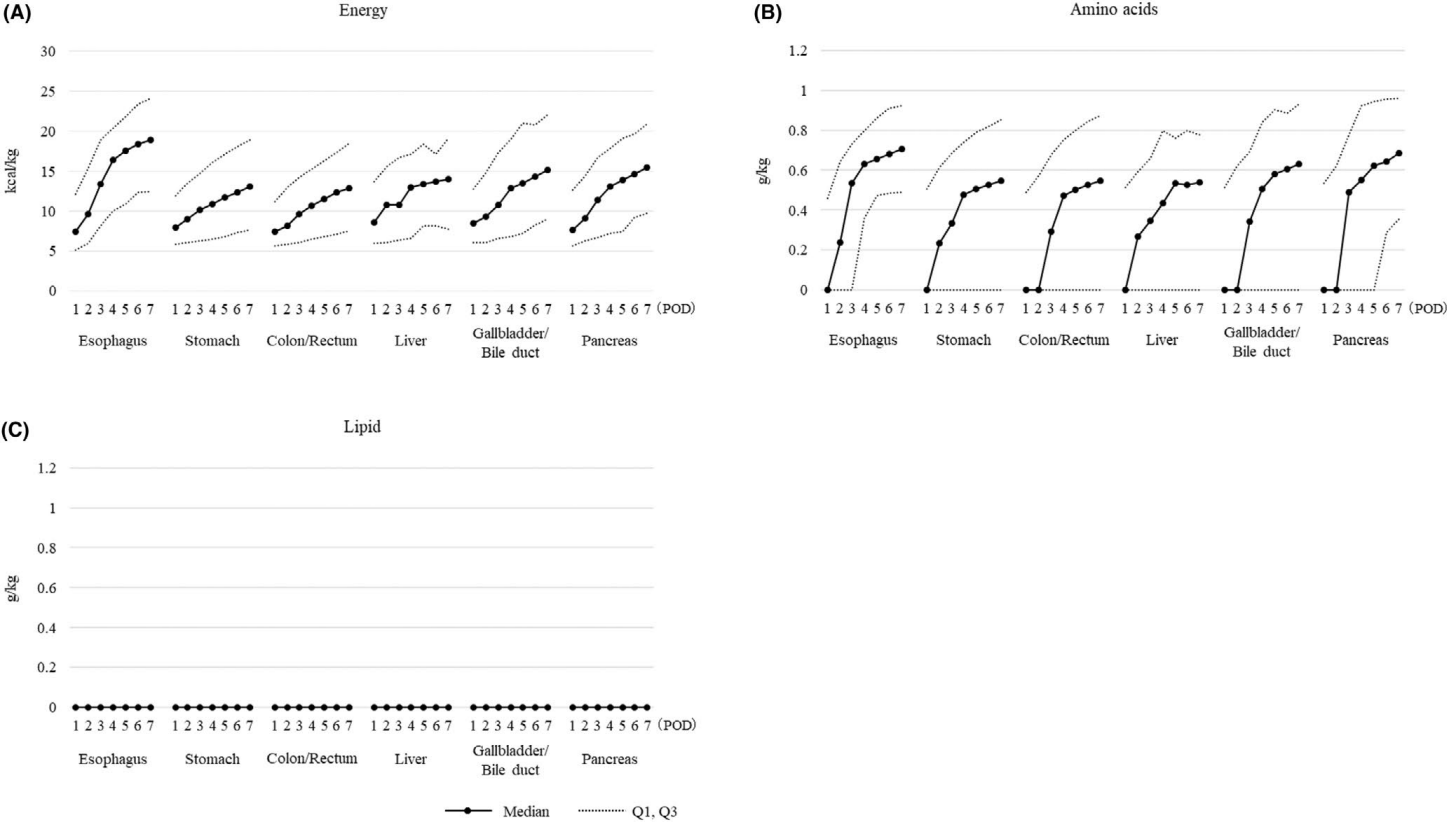
Prescribed parenteral (A) energy, (B) amino acid, and (C) lipid doses during postoperative d^a^ 1–7 among 19 145 adult patients in Japan who were fasting for 7 d or longer after gastroenterological cancer surgery from 2011 to 2022, by surgical site.^b^ Solid lines represent median doses, and dotted lines represent Quartile 1 (Q1) and Quartile 3 (Q3) doses. ^a^Postoperative day (POD) 1 defined as the next day after surgery. ^b^Groups (with numbers of patients) based on surgical sites: Esophagus (*n* = 1503); stomach (*n* = 5462); colon/rectum (*n* = 9967); liver (*n* = 151); gallbladder/bile duct (*n* = 407); and pancreas (*n* = 1655).

A total of 19 145 (5.3%) patients were fasting ≥7 d after surgery. The doses of parenteral energy, amino acids, and lipid prescribed to these patients on POD1 through POD7 are shown in Figure 5 and Table S6. On POD7, the median (Q1, Q3) prescribed energy and amino acid doses, by surgical site, were: esophagus, 18.9 (12.4, 24.1) kcal/kg and 0.71 (0.49, 0.92) g/kg; stomach, 13.0 (7.7, 18.9) kcal/kg and 0.55 (0.00, 0.85) g/kg; colon/rectum, 12.9 (7.6, 18.5) kcal/kg and 0.55 (0.00, 0.87) g/kg; liver, 14.0 (7.7, 19.1) kcal/kg and 0.54 (0.00, 0.78) g/kg; gall bladder/bile duct, 15.1 (9.0, 22.0) kcal/kg and 0.63 (0.00, 0.93) g/kg; and pancreas, 15.5 (9.8, 20.9) kcal/kg and 0.68 (0.36, 0.96) g/kg; all respectively. The median (Q1, Q3) prescribed lipid doses were 0.00 (0.00, 0.00) g/kg for the patients in all surgical site groups.

**FIGURE 6:**
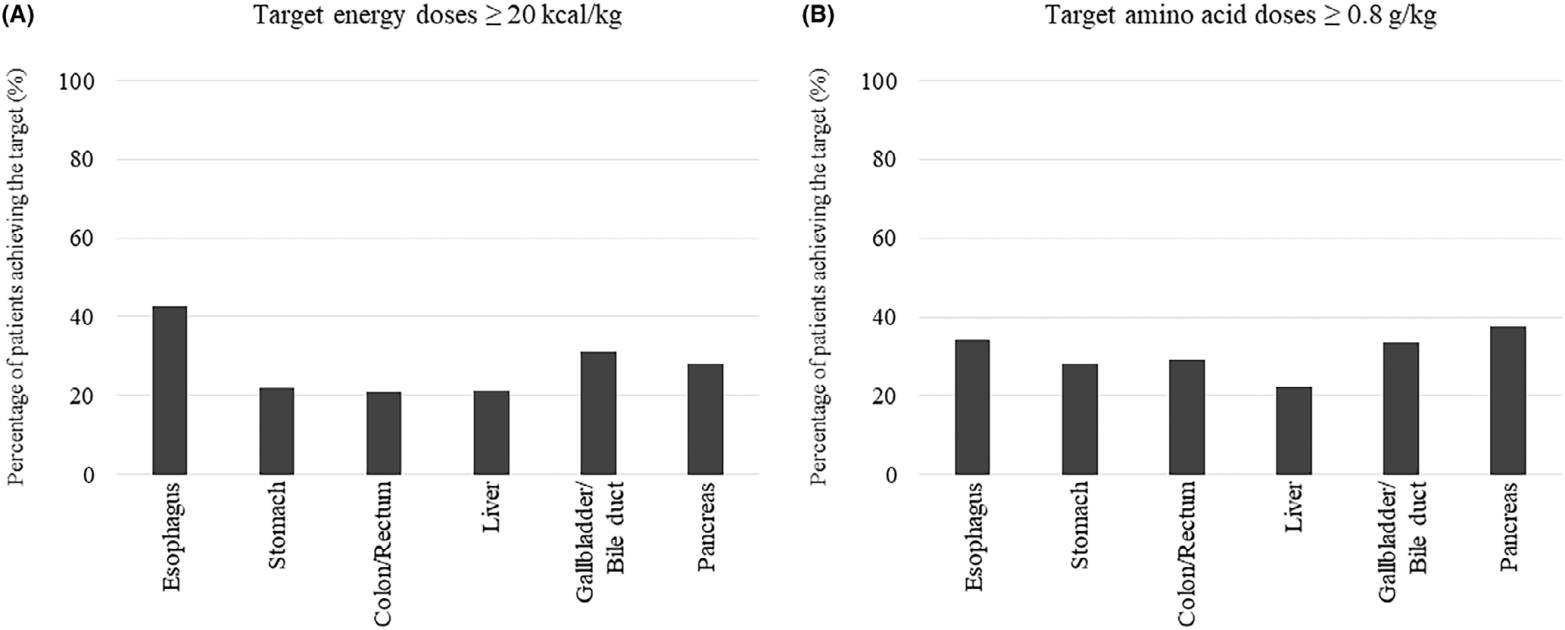
Patients receiving prescribed parenteral (A) energy and (B) amino acid target doses^a^ on postoperative d^b^ 7 among 19 145 adults in Japan who were fasting for 7 d or longer after gastroenterological cancer surgery from 2011 to 2022, by surgical site.^c a^Target parenteral energy (≥20 kcal/kg) and amino acid (≥0.8 g/kg) doses based on guideline recommendatons.^7^ ^b^Postoperative day 1 defined as the next day after surgery. ^c^Groups (with numbers of patients) based on surgical sites: Esophagus (*n* = 1503); stomach (*n* = 5462); colon/rectum (*n* = 9967); liver (*n* = 151); gallbladder/bile duct (*n* = 407); and pancreas (*n* = 1655).

The results regarding patients who were prescribed the guidelines‐based target parenteral energy and amino acid doses on POD7 are shown in Figure 6 and Table S7. The proportions of patients who were prescribed the target energy and amino acid doses, by surgical site, were: esophagus, 42.6% and 34.4%; stomach, 21.8% and 28.0%; colon/rectum, 20.9% and 29.1%; liver, 21.2% and 22.5%; gallbladder/bile duct, 31.0% and 33.4%; and pancreas, 28.2% and 37.8%; all respectively.

## 4 DISCUSSION

We investigated the nutritional management of patients during the 7 d after gastroenterological cancer surgery from 2011 to 2022 using the nationwide medical database. Of the patient groups based on the site of surgery for the GI tract, oral intake was started earliest in those undergoing liver surgery and latest in those undergoing esophageal surgery. During the postsurgical 7 d, most patients did not receive EN except those undergoing esophagus surgery, and approximately half of the patients in each surgical site group received SPN. This was administered to address energy and protein deficiencies despite the initiation of oral intake or EN. Finally, in patients fasting ≥7 d and receiving PN, the parenteral energy and amino acid doses prescribed within each surgical site group were insufficient, and almost no lipid was prescribed.

After gastroenterological surgery, oral intake was started on: POD2, liver surgery patients; POD3, colon/rectum and gallbladder/bile duct surgery patients, POD4, gastric and pancreatic surgery patients; POD7, esophageal surgery patients. A review article of patients undergoing esophageal surgery suggested that early initiation of oral intake led to higher risks of anastomotic leakage and aspiration pneumonia in 2019.^19^ However, a recent network meta‐analysis of patients undergoing upper GI cancer surgery reported that an early postoperative initiation of oral intake decreased the number of complications and enhanced postoperative recovery, without increasing the incidence of anastomotic leakage, in 2024.^20^ The same study suggested that the optimal time to resume oral intake for patients after upper GI surgery was POD3. Indeed, a quarter of the patients undergoing esophageal surgery in our study started oral intake on POD3. We found that open surgery, higher prescribed doses of crystalloid, colloid, albumin, and transfusion on the surgery day were independent predictive factors for prolonged postoperative fasting (ie, ≥7 d). Some studies reported that laparoscopic surgery was associated with the early postoperative oral intake by decreasing the surgical stress and promote the improvement of postoperative gastrointestinal functions.^21^, ^22^ In our study, the proportion of patients who initiated oral intake on POD3 was higher in the patients who underwent laparoscopic surgery than in those who did not for every surgical site. A study reported that an excessive fluid infusion load during surgery causes edema of the digestive tract, which may in turn lead to a delay in the recovery of GI function and a decrease in the strength of anastomoses.^23^ Our results support using laparoscopic surgery and to avoid the high amount of fluid infusion and transfusion on the day of gastroenterological cancer surgery. Although the initiation of oral intake may be late in Japan, approximately half of the patients started early oral intake after gastroenterological cancer surgery except for patients undergoing esophageal surgery.

Early oral intake or EN after surgery is recommended in Japanese and European nutritional guidelines.^7^, ^8^ We observed that in Japan, few patients undergoing gastroenterological cancer surgery excepting esophageal surgery, received early EN. On the other hand, many patients had PN prescribed during the postsurgical 7 d, and approximately half of patients had SPN prescribed (ie, PN along with oral intake or EN, to address energy and protein deficiencies) during that same period. Given those results, and the fact that oral intake was initiated by POD7 in 90% of all study patients (except for patients undergoing esophageal surgery), standard postoperative nutritional management in the patients in the study involved either early oral intake, PN, or SPN, but not EN. Regarding the use of SPN, a multicenter randomized controlled trial of patients undergoing major abdominal surgery reported that the implementation of SPN starting on POD3 resulted in patients achieving energy and protein intake targets, as well as a lower incidence of infectious complications.^24^ Furthermore, another study involving patients after major abdominal surgery reported that target energy and protein intake could not be achieved with early oral intake based on ERAS and nutritional guidelines.^25^ Despite this, SPN implementation before POD7 is not consistently recommended in nutritional guidelines in Europe and the United States.^8^, ^26^ Our study suggests that many patients undergoing gastroenterological cancer surgery receive SPN during the first postoperative week, and other studies indicate clinical benefits from early SPN, but further evaluation of the optimal time to initiate SPN and the appropriate duration of treatment is required.

Some patients undergoing GI cancer surgery are managed by PN alone because they cannot receive early initiation of oral intake or EN due to severe complications or GI intolerance.^4^ In our study, the proportion of patients who received PN alone by POD7 was relatively low: almost zero in those undergoing liver surgery, and about 10% in those undergoing surgery elsewhere in the GI tract. Although the prescribed doses of parenteral energy and amino acids for those who were fasting ≥7 d after surgery gradually increased from POD1 to POD7, the proportion of patients who were actually prescribed guidelines‐based target energy (20 kcal/kg) and amino acid (0.8 g/kg) doses was only 20%–40%. Patients need supplemental energy and amino acids after surgery because body protein breakdown is accelerated during postoperative stress.^27^ In addition, insufficient nutritional intake after GI cancer surgery has been reported to cause loss of body weight and muscle mass, as well as immune suppression, which in turn lead to increases in postoperative complications, prolongation of hospitalizations, and higher mortality rates.^28^, ^29^ Because patients who cannot receive oral intake or EN after GI cancer surgery are likely to have a high risk of malnutrition, they are expected to have a strictly‐controlled PN management with sufficient energy, amino acid, and lipid doses.

In this study, the proportions of patients who did not receive either oral intake or EN (ie, managed by infusions of either amino acids and lipid or carbohydrates and electrolytes) on POD3 after surgery of the stomach, colon/rectum, gallbladder/bile duct, or pancreas ranged from 40% and 60%. And, of the patients who received only infusions on POD3, half received infusions that contained only carbohydrate and electrolytes, without amino acids and lipid. It is unclear why many patients in our study received infusions containing only carbohydrate and electrolytes on POD3, and why many patients received insufficient doses of parenteral energy, amino acids, and lipid in the PN management. One possible reason, noted by others, is that surgeons tend to have the responsibility for implementing PN, yet often spend the majority of their time performing surgery and managing general perioperative care.^30^, ^31^ As a result, they may not have the time to deal with PN or a full awareness and understanding of guidelines‐based PN dose targets. However, a study showed evidence that in hospitalized patients managed with PN alone, those who were prescribed target energy, amino acids, and lipid doses experienced a mitigation of the decline in their performance of activities of daily living and an improvement in their mortality rates.^32^ In Japan, the goal of the earlier delivery of PN that contains target doses of energy, amino acids, and lipid to more patients after gastroenterological cancer surgery may be achieved by increasing surgeon awareness and multidisciplinary team (involving registered dietitians, nurses, and pharmacists) support for guidelines‐based nutritional management.

### 4.1 Study limitations

This study has several limitations. In the database used, there was no information available about the reasons why certain feeding routes were selected. As such, we were unable to assess whether appropriate feeding routes had been selected. Similarly, there was no data available in the database concerning the amounts of energy, amino acids, and lipid that were prescribed to patients who received oral intake or EN. Finally, the database did not include information on the amounts of PN discarded, so the doses of parenteral energy, amino acids, and lipid that fasting patients actually received may have been lower than the prescribed doses used for the study. Nonetheless, the study used real‐world data to evaluate the nutritional management of patients who underwent gastroenterological cancer surgery and accounted for 27% of the acute care hospitals in Japan. The results provide important insights into the current status of nutritional management in these patients, as well as potential improvements for postoperative nutritional management. A prospective study that includes some of the information missing in the database is needed in the future.

## 5 CONCLUSION

Oral intake after gastroenterological cancer surgery was started earliest in patients undergoing liver surgery and latest in those undergoing esophageal surgery. Few patients having gastroenterological cancer surgery received EN, aside from those who underwent esophageal surgery. SPN was prescribed to approximately half of all patients, regardless of their site of surgery. Parenteral energy and amino acid doses prescribed to fasting patients were insufficient, and almost no parenteral lipid was prescribed. Target energy and amino acid doses were not prescribed to more than half of fasting patients. The early initiation of oral intake and the use of guidelines‐based PN dosing in patients after gastroenterological cancer surgery should be further promoted. The use of SPN in these patients is feasible, while the optimal time to initiate SPN and appropriate duration of treatment need further evaluation.

## AUTHOR CONTRIBUTIONS

**Yoshikuni Kawaguchi:** Conceptualization; methodology; project administration; supervision; writing – original draft; writing – review and editing. **Kenta Murotani:** Conceptualization; methodology; writing – original draft; writing – review and editing. **Nahoki Hayashi:** Conceptualization; investigation; methodology; visualization; writing – original draft; writing – review and editing. **Satoru Kamoshita:** Conceptualization; investigation; methodology; project administration; supervision; visualization; writing – original draft; writing – review and editing.

## FUNDING INFORMATION

This study received no funding.

## CONFLICT OF INTEREST STATEMENT

Y. Kawaguchi received adviser and lecture fees from Otsuka Pharmaceutical Factory, Inc. K. Murotani received adviser fees from Otsuka Pharmaceutical Factory, Inc. N. Hayashi and S. Kamoshita were employed by Otsuka Pharmaceutical Factory, Inc.

## ETHICS STATEMENT

Approval of the research protocol by an Institutional Reviewer Board: The study was conducted after approval by the Shiba Palace Clinic Ethics Review Committee (Approval number, 153969_rn‐36 564), and it was registered with the University Hospital Medical Information Network (UMIN).

Informed Consent: An opt‐out for the study was not implemented because personal patient and hospital information obtained from the database were anonymized and there was no correspondence table. The article complies with the applicable guidelines outlined in the STrengthening the Reporting of OBservational studies in Epidemiology (STROBE) statement^33^ and in the REporting of studies Conducted using Observational Routinely‐collected health Data (RECORD) statement.^34^

Registry and the Registration No. of the study/trial: Clinical Trial Registry (registration number, UMIN000053645).

Animal Studies: N/A.

## Supporting information


**Table S1:** Japan‐specific medical claims codes.
**Table S2:** Feeding routes during postoperative d 1–7.
**Table S3:** Feeding routes during postoperative d 1–3, 1–5, and 1–7.
**Table S4:** Postoperative day of initiation of oral intake.
**Table S5:** Risk factors for postoperative fasting of 7 d or longer.
**Table S6:** Prescribed parenteral energy, amino acid, and lipid doses during postoperative d 1–7.
**Table S7:** Patients receiving prescribed parenteral energy and amino acid target doses on postoperative d7.

Appendix S1:

**Figure S1:** Feeding routes during postoperative d 1–7 with/without laparoscopic surgery.

**Figure S2:** Postoperative day of initiation of oral intake with/without postoperative complications.

## Data Availability

If requested, the authors will provide the data or will cooperate fully in obtaining and providing the data on which the article is based for examination by the editors or their assignees.
