# Supplementary material for: Nationwide medical database study for postoperative nutritional management in patients undergoing gastroenterological cancer surgery
Source: Ann Gastroenterol Surg. 2024 Nov 27;9(3):595–607. doi: 10.1002/ags3.12892 (PMC12080195; doi:10.1002/ags3.12892)
Supplement: Supplementary file 1 — Table S1: Japan‐specific medical claims codes. Table S2: Feeding routes during postoperative d 1–7. Table S3: Feeding routes during postoperative d 1–3, 1–5, and 1–7. Table S4: Postoperative day of initiation of oral intake. Table S5: Risk factors for postoperative fasting of 7 d or longer. Table S6: Prescribed parenteral energy, amino acid, and lipid doses during postoperative d 1–7. Table S7: Patients receiving prescribed parenteral energy and amino acid target doses on postoperative d7. [file AGS3-9-595-s001.docx]

**Supplementary Table S1. Japan-specific medical claims codes for 360,296 adult patients who underwent gastroenterological cancer surgery from 2011 to 2022**

| **Classification** | **Location / Type** | **Medical Claims Codes** |
| --- | --- | --- |
| **Surgical site** | Esophagus | K526, K526-2, K526-3, K526-4, K529-2, K529-3, K525, K526, K527, K527-2, K529, K529-4 |
|  | Stomach | K653, K653-4, K654-2, K654-3, K655, K655-2, K655-4, K655-5, K657, K657-2 |
|  | Colon/Rectum | K719, K719-2, K719-3, K720, K721-4, K739, K739-2, K739-3, K740, K740-2, K740-3 |
|  | Liver | K695, K695-2, K697-2, K697-3 |
|  | Gallbladder/Bile duct | K672, K672-2, K675, K675-2, K677, K677-2 |
|  | Pancreas | K700, K700-2, K700-3, K702, K702-2, K703, K703-2, K704 |
| **Type of cancer** | Esophagus | C150, C151, C152, C153, C154, C155, C158, C159 |
|  | Stomach | C160, C161, C162, C163, C164, C165, C166, C169 |
|  | Colon/Rectum | C182, C183, C184, C185, C186, C187, C189, C19, C20 |
|  | Liver | C220, C221, C222, C223, C224, C227, C229 |
|  | Gallbladder/Bile duct | C23, C240, C248, C249 |
|  | Pancreas | C250, C251, C252, C253, C254, C257, C258, C259 |
| **General anesthesia** |  | L001, L001-2, L007, L008, L009, L010 |
| **Preoperative oral management^†,^****^‡^** |  | 150371690 |
| **Preoperative cancer treatment^§^** | Chemotherapy | L01A0, L01B0, L01C1, L01C2, L01C3, L01C4, L01D0, L01F0, L01G1, L01G2, L01G3, L01G4, L01G5, L01G9, L01H1, L01H2, L01H3, L01H4, L01H5, L01H6, L01H9, L01J0, L01K0, L01L0, L01X3, L01X5, L01X8, L01X9 |
|  | Radiation therapy | M000, M000-2, M001, M001-2, M001-3, M001-4, M001-5, M002, M003, M004, M005 |
| **Prescribed infusions on day of surgery** | Crystalloid fluid | K01A7, K01A9, K01B1 |
|  | Colloid fluid^¶^ | K02A1, K02B0 |
|  | Albumin | K03B2, K03B3 |
|  | Transfusion^††^ | B02D6, B02D8, K03A0, K920, K920-2 |
| **Feeding routes^‡‡^** | Oral intake | 197000110, 197000710, 197001310, 197001710, V06B0 |
|  | Enteral nutrition | 140023210, 140057070, 140023350, 197003110, 197003210, 197003510 |
|  | Parenteral nutrition | K01D1, K01D2, K01E1, K01E2, K01E3, K01E4, K01E5 |
| **Intensive care unit admission** |  | A301, A301-2, A301-3, A301-4 |

^†^ Received from the day of hospital admission through the day before surgery.

^‡^ Support for oral intake functions, including swallowing and chewing.

^§^ Received from 60 days before surgery through the day before surgery.

^¶^ Prescribed dextrans and/or hydroxyethyl starches.

^††^ Prescribed blood cells, platelets, and/or fresh frozen plasma.

^‡‡^ For feeding routes: oral intake defined as meals served; enteral nutrition defined as tube feedings prescribed; parenteral nutrition defined as intravenous solutions containing amino acids and/or lipid prescribed.

**Supplementary Table S2. Feeding routes^†^ during postoperative days^‡^ 1–7, received by 360,296 adult patients in Japan who underwent gastroenterological cancer surgery from 2011 to 2022, by surgical site**

| **Postoperative Day** | **Feeding routes** | **Esophagus** | **Stomach** | **Colon/**  **Rectum** | **Liver** | **Gallbladder/**  **Bile duct** | **Pancreas** |
| --- | --- | --- | --- | --- | --- | --- | --- |
|  |  | N = 14,784 | N = 103,339 | N = 194,049 | N = 19,277 | N = 8,279 | N = 20,568 |
|  |  | n (%) | n (%) | n (%) | n (%) | n (%) | n (%) |
| **1** | Only oral intake | 534 (3.6) | 2,025 (2.0) | 4,961 (2.6) | 3,653 (19.0) | 1,048 (12.7) | 669 (3.3) |
|  | Oral intake + EN | 547 (3.7) | 70 (0.1) | 61 (0.0) | 18 (0.1) | 6 (0.1) | 72 (0.4) |
|  | Oral intake + PN | 316 (2.1) | 2,731 (2.6) | 3,527 (1.8) | 948 (4.9) | 318 (3.8) | 449 (2.2) |
|  | Oral intake + EN + PN | 218 (1.5) | 52 (0.1) | 12 (0.0) | 4 (0.0) | 3 (0.0) | 11 (0.1) |
|  | Only EN | 3,328 (22.5) | 749 (0.7) | 1,145 (0.6) | 103 (0.5) | 127 (1.5) | 552 (2.7) |
|  | EN + PN | 1,503 (10.2) | 547 (0.5) | 583 (0.3) | 12 (0.1) | 40 (0.5) | 221 (1.1) |
|  | Only PN | 2,894 (19.6) | 41,429 (40.1) | 70,418 (36.3) | 4,095 (21.2) | 2,158 (26.1) | 6,301 (30.6) |
|  | Other | 5,444 (36.8) | 55,736 (53.9) | 113,342 (58.4) | 10,444 (54.2) | 4,579 (55.3) | 12,293 (59.8) |
| **2** | Only oral intake | 1,035 (7.0) | 7,836 (7.6) | 22,533 (11.6) | 9,499 (49.3) | 2,149 (26.0) | 1,547 (7.5) |
|  | Oral intake + EN | 592 (4.0) | 191 (0.2) | 182 (0.1) | 31 (0.2) | 39 (0.5) | 112 (0.5) |
|  | Oral intake + PN | 541 (3.7) | 8,267 (8.0) | 18,887 (9.7) | 3,288 (17.1) | 940 (11.4) | 1,285 (6.2) |
|  | Oral intake + EN + PN | 522 (3.5) | 190 (0.2) | 113 (0.1) | 11 (0.1) | 22 (0.3) | 58 (0.3) |
|  | Only EN | 3,592 (24.3) | 874 (0.8) | 1,706 (0.9) | 113 (0.6) | 205 (2.5) | 681 (3.3) |
|  | EN + PN | 2,651 (17.9) | 917 (0.9) | 2,374 (1.2) | 26 (0.1) | 139 (1.7) | 1,026 (5.0) |
|  | Only PN | 3,005 (20.3) | 42,275 (40.9) | 66,586 (34.3) | 2,320 (12.0) | 2,093 (25.3) | 6,821 (33.2) |
|  | Other | 2,846 (19.3) | 42,789 (41.4) | 81,668 (42.1) | 3,989 (20.7) | 2,692 (32.5) | 9,038 (43.9) |
| **3** | Only oral intake | 1,321 (8.9) | 21,497 (20.8) | 55,037 (28.4) | 11,942 (61.9) | 3,145 (38.0) | 3,789 (18.4) |
|  | Oral intake + EN | 497 (3.4) | 297 (0.3) | 509 (0.3) | 44 (0.2) | 61 (0.7) | 181 (0.9) |
|  | Oral intake + PN | 834 (5.6) | 21,599 (20.9) | 41,238 (21.3) | 4,804 (24.9) | 1,602 (19.4) | 3,011 (14.6) |
|  | Oral intake + EN + PN | 699 (4.7) | 377 (0.4) | 505 (0.3) | 18 (0.1) | 44 (0.5) | 199 (1.0) |
|  | Only EN | 3,306 (22.4) | 672 (0.7) | 903 (0.5) | 38 (0.2) | 191 (2.3) | 585 (2.8) |
|  | EN + PN | 3,646 (24.7) | 804 (0.8) | 1,097 (0.6) | 33 (0.2) | 158 (1.9) | 1,111 (5.4) |
|  | Only PN | 3,038 (20.5) | 30,433 (29.4) | 48,290 (24.9) | 981 (5.1) | 1,661 (20.1) | 6,159 (29.9) |
|  | Other | 1,443 (9.8) | 27,660 (26.8) | 46,470 (23.9) | 1,417 (7.4) | 1,417 (17.1) | 5,533 (26.9) |
| **4** | Only oral intake | 1,543 (10.4) | 37,208 (36.0) | 82,682 (42.6) | 14,011 (72.7) | 3,879 (46.9) | 5,874 (28.6) |
|  | Oral intake + EN | 522 (3.5) | 386 (0.4) | 379 (0.2) | 32 (0.2) | 81 (1.0) | 250 (1.2) |
|  | Oral intake + PN | 1,017 (6.9) | 31,229 (30.2) | 53,239 (27.4) | 3,964 (20.6) | 2,047 (24.7) | 4,973 (24.2) |
|  | Oral intake + EN + PN | 821 (5.6) | 379 (0.4) | 400 (0.2) | 21 (0.1) | 78 (0.9) | 559 (2.7) |
|  | Only EN | 3,090 (20.9) | 539 (0.5) | 814 (0.4) | 44 (0.2) | 153 (1.8) | 489 (2.4) |
|  | EN + PN | 3,998 (27.0) | 867 (0.8) | 963 (0.5) | 33 (0.2) | 164 (2.0) | 846 (4.1) |
|  | Only PN | 2,909 (19.7) | 19,674 (19.0) | 32,322 (16.7) | 624 (3.2) | 1,146 (13.8) | 4,634 (22.5) |
|  | Other | 884 (6.0) | 13,057 (12.6) | 23,250 (12.0) | 548 (2.8) | 731 (8.8) | 2,943 (14.3) |
| **5** | Only oral intake | 1,757 (11.9) | 49,325 (47.7) | 107,116 (55.2) | 15,130 (78.5) | 4,382 (52.9) | 7,344 (35.7) |
|  | Oral intake + EN | 588 (4.0) | 425 (0.4) | 354 (0.2) | 40 (0.2) | 96 (1.2) | 377 (1.8) |
|  | Oral intake + PN | 1,186 (8.0) | 32,402 (31.4) | 49,753 (25.6) | 3,203 (16.6) | 2,164 (26.1) | 6,117 (29.7) |
|  | Oral intake + EN + PN | 839 (5.7) | 447 (0.4) | 409 (0.2) | 17 (0.1) | 103 (1.2) | 792 (3.9) |
|  | Only EN | 2,994 (20.3) | 452 (0.4) | 508 (0.3) | 41 (0.2) | 122 (1.5) | 351 (1.7) |
|  | EN + PN | 4,136 (28.0) | 768 (0.7) | 737 (0.4) | 41 (0.2) | 149 (1.8) | 592 (2.9) |
|  | Only PN | 2,681 (18.1) | 12,653 (12.2) | 22,223 (11.5) | 464 (2.4) | 852 (10.3) | 3,354 (16.3) |
|  | Other | 603 (4.1) | 6,867 (6.6) | 12,949 (6.7) | 341 (1.8) | 411 (5.0) | 1,641 (8.0) |
| **6** | Only oral intake | 1,989 (13.5) | 60,494 (58.5) | 127,358 (65.6) | 16,212 (84.1) | 4,832 (58.4) | 8,414 (40.9) |
|  | Oral intake + EN | 653 (4.4) | 474 (0.5) | 261 (0.1) | 38 (0.2) | 111 (1.3) | 607 (3.0) |
|  | Oral intake + PN | 1,439 (9.7) | 27,526 (26.6) | 37,975 (19.6) | 2,307 (12.0) | 2,036 (24.6) | 6,488 (31.5) |
|  | Oral intake + EN + PN | 979 (6.6) | 441 (0.4) | 274 (0.1) | 20 (0.1) | 107 (1.3) | 637 (3.1) |
|  | Only EN | 3,050 (20.6) | 376 (0.4) | 389 (0.2) | 42 (0.2) | 103 (1.2) | 301 (1.5) |
|  | EN + PN | 3,841 (26.0) | 662 (0.6) | 625 (0.3) | 35 (0.2) | 139 (1.7) | 486 (2.4) |
|  | Only PN | 2,311 (15.6) | 9,171 (8.9) | 17,993 (9.3) | 371 (1.9) | 650 (7.9) | 2,673 (13.0) |
|  | Other | 5,22 (3.5) | 4,195 (4.1) | 9,174 (4.7) | 252 (1.3) | 301 (3.6) | 962 (4.7) |
| **7** | Only oral intake | 2,430 (16.4) | 68,871 (66.6) | 141,685 (73.0) | 16,910 (87.7) | 5,236 (63.2) | 9,570 (46.5) |
|  | Oral intake + EN | 1,317 (8.9) | 523 (0.5) | 219 (0.1) | 37 (0.2) | 134 (1.6) | 730 (3.5) |
|  | Oral intake + PN | 1,959 (13.3) | 23,433 (22.7) | 30,973 (16.0) | 1,734 (9.0) | 1,857 (22.4) | 6,504 (31.6) |
|  | Oral intake + EN + PN | 1,447 (9.8) | 450 (0.4) | 301 (0.2) | 16 (0.1) | 112 (1.4) | 594 (2.9) |
|  | Only EN | 2,607 (17.6) | 334 (0.3) | 376 (0.2) | 45 (0.2) | 82 (1.0) | 209 (1.0) |
|  | EN + PN | 2,903 (19.6) | 577 (0.6) | 544 (0.3) | 45 (0.2) | 118 (1.4) | 394 (1.9) |
|  | Only PN | 1,726 (11.7) | 6,496 (6.3) | 13,615 (7.0) | 306 (1.6) | 504 (6.1) | 1,952 (9.5) |
|  | Other | 395 (2.7) | 2,655 (2.6) | 6,336 (3.3) | 184 (1.0) | 236 (2.9) | 615 (3.0) |

^†^ For feeding routes: oral intake defined as meals served; enteral nutrition (EN) defined as tube feedings prescribed; parenteral nutrition (PN) defined as intravenous solutions containing amino acids and/or lipid prescribed; and other defined as intravenous solutions containing only glucose and electrolytes prescribed.

^‡^ Postoperative day 1 defined as the next day after surgery.

**Supplementary Table S3. Feeding routes^†^ during postoperative days^‡^ 1–3, 1–5, and 1–7, received by 360,296 adult patients in Japan who underwent gastroenterological cancer surgery from 2011 to 2022, by surgical site**

| **Postoperative Days** | **Feeding routes** | **Esophagus** | **Stomach** | **Colon/**  **Rectum** | **Liver** | **Gallbladder/**  **Bile duct** | **Pancreas** |
| --- | --- | --- | --- | --- | --- | --- | --- |
|  |  | N = 14,784 | N = 103,339 | N = 194,049 | N = 19,277 | N = 8,279 | N = 20,568 |
|  |  | n (%) | n (%) | n (%) | n (%) | n (%) | n (%) |
| **1–3** | Oral intake | 3,987 (27.0) | 44,940 (43.5) | 100,312 (51.7) | 16,977 (88.1) | 5,024 (60.7) | 7,727 (37.6) |
|  | EN | 8,579 (58.0) | 2,884 (2.8) | 5,620 (2.9) | 270 (1.4) | 547 (6.6) | 2,440 (11.9) |
|  | PN | 8,629 (58.4) | 56,099 (54.3) | 96,805 (49.9) | 6,648 (34.5) | 3,781 (45.7) | 10,881 (52.9) |
|  | SPN | 5,568 (37.7) | 24,878 (24.1) | 46,779 (24.1) | 5,482 (28.4) | 2,086 (25.2) | 4,576 (22.2) |
| **1–5** | Oral intake | 5,369 (36.3) | 85,370 (82.6) | 166,070 (85.6) | 18,790 (97.5) | 7,059 (85.3) | 15,276 (74.3) |
|  | EN | 9,294 (62.9) | 3,740 (3.6) | 6,866 (3.5) | 334 (1.7) | 674 (8.1) | 2,851 (13.9) |
|  | PN | 9,801 (66.3) | 59,688 (57.8) | 104,385 (53.8) | 7,191 (37.3) | 4,305 (52.0) | 12,304 (59.8) |
|  | SPN | 7,218 (48.8) | 46,438 (44.9) | 81,222 (41.9) | 6,592 (34.2) | 3,461 (41.8) | 8,909 (43.3) |
| **1–7** | Oral intake | 8,303 (56.2) | 97,128 (94.0) | 183,141 (94.4) | 19,087 (99.0) | 7,718 (93.2) | 18,421 (89.6) |
|  | EN | 9,573 (64.8) | 4,158 (4.0) | 7,400 (3.8) | 373 (1.9) | 741 (9.0) | 3,047 (14.8) |
|  | PN | 10,113 (68.4) | 61,415 (59.4) | 107,479 (55.4) | 7,369 (38.2) | 4,499 (54.3) | 12,946 (62.9) |
|  | SPN | 8,488 (57.4) | 54,669 (52.9) | 93,542 (48.2) | 6,944 (36.0) | 4,022 (48.6) | 11,170 (54.3) |

^†^ For feeding routes: oral intake defined as meals served; enteral nutrition (EN) defined as tube feedings prescribed; parenteral nutrition (PN) defined as intravenous solutions containing amino acids and/or lipid prescribed; and supplemental parenteral nutrition (SPN) defined as PN prescribed on same day as oral intake and/or EN.

^‡^ Postoperative day 1 defined as the next day after surgery.

**Supplementary Table S4. Postoperative day^†^ of initiation of oral intake among 359,138 adult patients in Japan who underwent gastroenterological cancer surgery and initiated oral intake during the hospitalized period from 2011 to 2022, by surgical site**

| ***Postoperative Day of Oral Intake Initiated*** | **Esophagus** | **Stomach** | **Colon/**  **Rectum** | **Liver** | **Gallbladder/**  **Bile duct** | **Pancreas** |
| --- | --- | --- | --- | --- | --- | --- |
|  | N = 14,537 | N = 103,052 | N = 193,532 | N = 19,257 | N = 8,229 | N = 20,531 |
| **POD, median (Q1, Q3)** | 7 (3, 10) | 4 (3, 5) | 3 (3, 5) | 2 (2, 3) | 3 (2, 4) | 4 (3, 6) |
| **POD, n (%)** | | | | | | |
| **1** | 1,615 (11.1) | 4,878 (4.7) | 8,561 (4.4) | 4,623 (24.0) | 1,375 (16.7) | 1,201 (5.8) |
| **2** | 1,475 (10.1) | 12,261 (11.9) | 34,016 (17.6) | 8,262 (42.9) | 1,855 (22.5) | 2,203 (10.7) |
| **3** | 897 (6.2) | 27,801 (27.0) | 57,735 (29.8) | 4,092 (21.2) | 1,794 (21.8) | 4,323 (21.1) |
| **4** | 735 (5.1) | 26,225 (25.4) | 42,360 (21.9) | 1,380 (7.2) | 1,322 (16.1) | 4,515 (22.0) |
| **5** | 647 (4.5) | 14,205 (13.8) | 23,398 (12.1) | 433 (2.2) | 713 (8.7) | 3,034 (14.8) |
| **6** | 963 (6.6) | 7,028 (6.8) | 9,712 (5.0) | 199 (1.0) | 391 (4.8) | 1,721 (8.4) |
| **7** | 1,971 (13.6) | 4,730 (4.6) | 7,359 (3.8) | 98 (0.5) | 268 (3.3) | 1,424 (6.9) |
| **> 7** | 6,234 (42.9) | 5,924 (5.7) | 10,391 (5.4) | 170 (0.9) | 511 (6.2) | 2,110 (10.3) |

^†^ Postoperative day (POD) 1 defined as the next day after surgery.

**Abbreviations**: Q1, quartile 1; Q3, quartile 3.

**Supplementary Table S5. Risk factors for postoperative fasting of 7 days or longer among 360,296 adult patients in Japan who underwent gastroenterological cancer surgery from 2011 to 2022**

| **Patient Characteristics** | ***Variables*** | **Non-adjusted OR (95% CI)** | **Adjusted^†^ OR (95% CI)** |
| --- | --- | --- | --- |
| **Age**, *yrs* | 18–59 | Reference | Reference |
|  | 60–69 | 1.07 (1.02–1.12) | 0.98 (0.93–1.03) |
|  | 70–79 | 1.08 (1.03–1.13) | 1.01 (0.96–1.06) |
|  | 80–89 | 1.15 (1.09–1.21) | **1.08 (1.03–1.14)** |
|  | ≥ 90 | 1.21 (1.08–1.35) | 1.04 (0.92–1.17) |
| **Sex** | Male | Reference | Reference |
|  | Female | 0.53 (0.51–0.55) | **0.55 (0.53–0.57)** |
| **BMI** | < 16 | 1.27 (1.16–1.39) | 1.02 (0.92–1.14) |
|  | ≥ 16, < 18.5 | 1.05 (1.00–1.10) | 0.96 (0.89–1.03) |
|  | ≥ 18.5, < 22.5 | Reference | Reference |
|  | ≥ 22.5, < 25 | 1.01 (0.98–1.05) | **1.06 (1.01–1.10)** |
|  | ≥ 25, < 30 | 1.05 (1.01–1.10) | **1.16 (1.11–1.21)** |
|  | ≥ 30 | 1.07 (0.99–1.17) | **1.30 (1.19–1.42)** |
| **Beds in admission hospital** | < 200 | Reference | Reference |
|  | ≥ 200, < 500 | 0.79 (0.74–0.84) | **0.81 (0.76–0.86)** |
|  | ≥ 500 | 0.70 (0.66–0.74) | **0.73 (0.69–0.78)** |
| **Admission type** | Elective | Reference | Reference |
|  | Emergency | 2.20 (2.11–2.29) | **1.30 (1.23–1.37)** |
|  | NA | 1.76 (1.66–1.86) | 1.17 (1.10–1.24) |
| **Charlson Comorbidity Index** | 0–1 | Reference | Reference |
|  | 2–3 | 0.75 (0.70–0.80) | **0.85 (0.80–0.91)** |
|  | 4–5 | 0.74 (0.68–0.80) | 0.94 (0.87–1.02) |
|  | ≥ 6 | 0.97 (0.90–1.05) | 0.92 (0.85–1.00) |
| **Barthel Index** | 100 | Reference | Reference |
|  | 65–95 | 1.24 (1.17–1.32) | **1.08 (1.01–1.16)** |
|  | 45–60 | 1.60 (1.46–1.74) | **1.21 (1.08–1.35)** |
|  | 5–40 | 1.98 (1.81–2.17) | **1.37 (1.21–1.56)** |
|  | 0 | 2.95 (2.73–3.18) | **1.71 (1.39–2.11)** |
|  | NA | 1.70 (1.58–1.83) | 1.18 (1.06–1.32) |
| **Smoking history** | Yes | 1.29 (1.25–1.33) | 1.03 (1.00–1.07) |
|  | No | Reference | Reference |
|  | NA | 1.45 (1.38–1.52) | 1.12 (1.06–1.18) |
| **Low BMI^‡^** | Yes | 1.07 (1.03–1.11) | 1.01 (0.95–1.07) |
|  | No | Reference | Reference |
| **TNM cancer classification** | Ⅰ | Reference | Reference |
|  | Ⅱ | 1.31 (1.25–1.38) | 1.02 (0.97–1.07) |
|  | Ⅲ | 1.42 (1.35–1.49) | 1.04 (0.99–1.10) |
|  | Ⅳ | 1.63 (1.53–1.73) | 0.99 (0.93–1.06) |
|  | NA | 1.66 (1.59–1.74) | 1.22 (1.16–1.28) |
| **Level of food intake independence** | Required no assistance | Reference | Reference |
|  | Required partial assistance | 1.66 (1.55–1.79) | 1.08 (0.97–1.21) |
|  | Required full assistance | 2.67 (2.50–2.87) | 1.02 (0.84–1.23) |
|  | NA | 1.99 (1.79–2.23) | 1.20 (1.03–1.40) |
| **Preoperative oral management^§,¶^** | Yes | Reference | Reference |
|  | No | 1.27 (1.22–1.31) | **1.14 (1.10–1.19)** |
| **Preoperative artificial nutrition^§^** | Enteral nutrition^††^ | 0.88 (0.79–0.97) | **0.63 (0.57–0.70)** |
|  | Parenteral nutrition^‡‡^ | 1.52 (1.47–1.57) | **1.22 (1.17–1.26)** |
| **Preoperative cancer treatments^§§^** | Chemotherapy | 1.34 (1.27–1.41) | **0.86 (0.82–0.92)** |
|  | Radiation therapy | 1.36 (1.21–1.52) | 1.05 (0.93–1.19) |
| **Surgical site** | Esophagus | 14.3 (12.1–17.0) | **18.8 (15.8–22.3)** |
|  | Stomach | 7.07 (6.01–8.31) | **9.71 (8.23–11.5)** |
|  | Colorectal | 6.86 (5.84–8.06) | **10.8 (9.12–12.7)** |
|  | Liver | Reference | Reference |
|  | Gallbladder/Bile duct | 6.55 (5.42-7.91) | **6.04 (4.99–7.30)** |
|  | Pancreas | 11.1 (9.37–13.1) | **11.0 (9.31–13.1)** |
| **Surgical methods** | Laparoscopic | Reference | Reference |
|  | Open | 2.01 (1.95–2.07) | **1.51 (1.45–1.56)** |
| **Prescribed infusions on day of surgery** | ***Crystalloid fluid (mL)*** | | |
|  | ≤ 5,000 | Reference | Reference |
|  | > 5,000, ≤ 10,000 | 1.73 (1.66–1.80) | **1.43 (1.37–1.49)** |
|  | > 10,000 | 3.55 (3.38–3.72) | **2.10 (1.99–2.21)** |
|  | ***Colloid fluid^¶¶^ (mL)*** | | |
|  | 0 | Reference | Reference |
|  | > 0, ≤ 500 | 1.24 (1.19–1.28) | **1.11 (1.07–1.15)** |
|  | > 500 | 1.92 (1.86–1.99) | **1.34 (1.29–1.39)** |
|  | ***Albumin (mL)*** | | |
|  | 0 | Reference | Reference |
|  | > 0, ≤ 500 | 1.92 (1.84–2.01) | **1.19 (1.13–1.25)** |
|  | > 500 | 2.93 (2.74–3.13) | **1.52 (1.41–1.64)** |
|  | ***Transfusion^†††^ (mL)*** | | |
|  | 0 | Reference | Reference |
|  | > 0, ≤ 500 | 1.63 (1.53–1.73) | **1.12 (1.06–1.20)** |
|  | > 500 | 2.40 (2.30–2.50) | **1.37 (1.30–1.44)** |
| **ICU admission on day of surgery** |  | 1.23 (1.20–1.27) | **1.07 (1.04–1.11)** |

^†^ Adjusted for age, sex, BMI, beds in admission hospital, admission type, Charlson Comorbidity Index, Barthel Index, smoking history, low BMI, TNM cancer classification, level of food intake independence, preoperative medical treatments (preoperative oral management, enteral nutrition, parenteral nutrition, chemotherapy, radiation therapy), surgical site, surgical methods, prescribed infusions on day of surgery (crystalloid fluid, colloid fluid, albumin, transfusion), and ICU admission on day of surgery.

^‡^ Low BMI defined as BMI < 18.5 in the patients < 70 years old and BMI < 20 in those ≥ 70 years old.

^§^ Received from the day of hospital admission through the day before surgery.

^¶^ Support for oral intake functions, including swallowing and chewing.

^††^ Enteral nutrition defined as tube feedings prescribed.

^‡‡^ Parenteral nutrition defined as intravenous solutions containing amino acids and/or lipid prescribed.

^§§^ Received from 60 days before surgery through the day before surgery.

^¶¶^ Prescribed dextrans and/or hydroxyethyl starches.

^†††^ Prescribed blood cells, platelets, and/or fresh frozen plasma.

**Abbreviations**: OR, odds ratio; CI, confidence interval; BMI, body mass index; NA, not available; ICU, intensive care unit.

**Supplementary Table S6. Prescribed parenteral energy, amino acid, and lipid doses during postoperative days^†^ 1–7 among 19,145 adult patients in Japan who were fasting for 7 days or longer after gastroenterological cancer surgery from 2011 to 2022, by surgical site**

| **Components** | ***Postoperative Day*** | **Esophagus** | **Stomach** | **Colon/**  **Rectum** | **Liver** | **Gallbladder/**  **Bile duct** | **Pancreas** | |
| --- | --- | --- | --- | --- | --- | --- | --- | --- |
|  |  | N = 1,503 | N = 5,462 | N = 9,967 | N = 151 | N = 407 | N = 1,655 | |
| ***Medians (Quartile 1, Quartile 3)*** | | | | | | | |  |
| **Energy (kcal/kg)** | 1 | 7.4 (5.1, 12.1) | 7.9 (5.8, 11.9) | 7.5 (5.7, 11.1) | 8.6 (6.0, 13.7) | 8.5 (6.1, 12.8) | 7.6 (5.7, 12.6) | |
|  | 2 | 9.7 (5.9, 15.3) | 9.0 (6.1, 13.5) | 8.2 (5.8, 12.9) | 10.8 (6.0, 15.6) | 9.3 (6.0, 14.9) | 9.1 (6.3, 14.4) | |
|  | 3 | 13.4 (8.1, 18.9) | 10.1 (6.3, 14.7) | 9.6 (6.1, 14.2) | 10.8 (6.3, 16.7) | 10.8 (6.6, 17.4) | 11.4 (6.7, 16.8) | |
|  | 4 | 16.4 (10.1, 20.4) | 10.9 (6.5, 16.0) | 10.7 (6.4, 15.3) | 13.0 (6.6, 17.1) | 12.9 (6.8, 19.0) | 13.1 (7.2, 17.8) | |
|  | 5 | 17.6 (10.9, 21.8) | 11.7 (6.8, 17.1) | 11.5 (6.8, 16.3) | 13.4 (8.2, 18.3) | 13.5 (7.2, 21.0) | 13.9 (7.4, 19.2) | |
|  | 6 | 18.3 (12.3, 23.4) | 12.3 (7.3, 18.1) | 12.3 (7.1, 17.4) | 13.7 (8.2, 17.2) | 14.3 (8.2, 20.8) | 14.6 (9.2, 19.6) | |
|  | 7 | 18.9 (12.4, 24.1) | 13.0 (7.7, 18.9) | 12.9 (7.6, 18.5) | 14.0 (7.7, 19.1) | 15.1 (9.0, 22.0) | 15.5 (9.8, 20.9) | |
| **Amino acids (g/kg)** | 1 | 0.00 (0.00, 0.46) | 0.00 (0.00, 0.51) | 0.00 (0.00, 0.49) | 0.00 (0.00, 0.51) | 0.00 (0.00, 0.51) | 0.00 (0.00, 0.53) | |
|  | 2 | 0.24 (0.00, 0.64) | 0.24 (0.00, 0.62) | 0.00 (0.00, 0.57) | 0.27 (0.00, 0.59) | 0.00 (0.00, 0.62) | 0.00 (0.00, 0.62) | |
|  | 3 | 0.53 (0.00, 0.73) | 0.33 (0.00, 0.68) | 0.30 (0.00, 0.68) | 0.35 (0.00, 0.66) | 0.34 (0.00, 0.69) | 0.49 (0.00, 0.78) | |
|  | 4 | 0.63 (0.36, 0.80) | 0.48 (0.00, 0.74) | 0.47 (0.00, 0.75) | 0.44 (0.00, 0.80) | 0.51 (0.00, 0.84) | 0.55 (0.00, 0.92) | |
|  | 5 | 0.66 (0.47, 0.86) | 0.51 (0.00, 0.79) | 0.50 (0.00, 0.80) | 0.53 (0.00, 0.76) | 0.58 (0.00, 0.90) | 0.62 (0.00, 0.94) | |
|  | 6 | 0.68 (0.48, 0.91) | 0.53 (0.00, 0.82) | 0.53 (0.00, 0.84) | 0.53 (0.00, 0.80) | 0.61 (0.00, 0.89) | 0.64 (0.29, 0.96) | |
|  | 7 | 0.71 (0.49, 0.92) | 0.55 (0.00, 0.85) | 0.55 (0.00, 0.87) | 0.54 (0.00, 0.78) | 0.63 (0.00, 0.93) | 0.68 (0.36, 0.96) | |
| **Lipid (g/kg)** | 1 | 0.00 (0.00, 0.00) | 0.00 (0.00, 0.00) | 0.00 (0.00, 0.00) | 0.00 (0.00, 0.00) | 0.00 (0.00, 0.00) | 0.00 (0.00, 0.00) | |
|  | 2 | 0.00 (0.00, 0.00) | 0.00 (0.00, 0.00) | 0.00 (0.00, 0.00) | 0.00 (0.00, 0.00) | 0.00 (0.00, 0.00) | 0.00 (0.00, 0.00) | |
|  | 3 | 0.00 (0.00, 0.00) | 0.00 (0.00, 0.00) | 0.00 (0.00, 0.00) | 0.00 (0.00, 0.00) | 0.00 (0.00, 0.00) | 0.00 (0.00, 0.00) | |
|  | 4 | 0.00 (0.00, 0.00) | 0.00 (0.00, 0.00) | 0.00 (0.00, 0.00) | 0.00 (0.00, 0.00) | 0.00 (0.00, 0.00) | 0.00 (0.00, 0.00) | |
|  | 5 | 0.00 (0.00, 0.00) | 0.00 (0.00, 0.00) | 0.00 (0.00, 0.00) | 0.00 (0.00, 0.00) | 0.00 (0.00, 0.00) | 0.00 (0.00, 0.00) | |
|  | 6 | 0.00 (0.00, 0.00) | 0.00 (0.00, 0.00) | 0.00 (0.00, 0.00) | 0.00 (0.00, 0.00) | 0.00 (0.00, 0.00) | 0.00 (0.00, 0.00) | |
|  | 7 | 0.00 (0.00, 0.00) | 0.00 (0.00, 0.00) | 0.00 (0.00, 0.00) | 0.00 (0.00, 0.00) | 0.00 (0.00, 0.00) | 0.00 (0.00, 0.00) | |
| ***Means (Standard Deviations)*** | | | | | | | |  |
| **Energy (kcal/kg)** | 1 | 8.8 (5.4) | 9.1 (4.9) | 8.8 (5.8) | 10.0 (5.3) | 9.6 (4.9) | 9.5 (5.3) | |
|  | 2 | 11.1 (6.5) | 10.2 (5.8) | 9.7 (5.6) | 11.3 (6.4) | 10.9 (6.3) | 10.8 (6.0) | |
|  | 3 | 14.1 (7.5) | 11.3 (6.5) | 10.9 (6.4) | 12.1 (7.0) | 12.4 (7.5) | 12.5 (7.0) | |
|  | 4 | 16.1 (8.6) | 12.2 (7.2) | 11.9 (7.0) | 13.3 (7.8) | 13.7 (8.6) | 13.7 (7.6) | |
|  | 5 | 17.2 (8.2) | 12.9 (7.6) | 12.7 (7.4) | 13.8 (7.7) | 14.8 (8.7) | 14.7 (9.1) | |
|  | 6 | 18.0 (8.5) | 13.6 (7.9) | 13.4 (7.8) | 13.7 (7.5) | 15.2 (8.4) | 15.4 (8.0) | |
|  | 7 | 18.2 (8.3) | 14.3 (8.2) | 14.1 (8.2) | 13.9 (7.9) | 16.0 (9.0) | 16.1 (8.2) | |
| **Amino acids (g/kg)** | 1 | 0.21 (0.34) | 0.26 (0.36) | 0.24 (0.35) | 0.27 (0.35) | 0.27 (0.38) | 0.27 (0.40) | |
|  | 2 | 0.35 (0.40) | 0.34 (0.40) | 0.32 (0.38) | 0.36 (0.44) | 0.32 (0.40) | 0.33 (0.40) | |
|  | 3 | 0.50 (0.41) | 0.39 (0.40) | 0.38 (0.40) | 0.41 (0.44) | 0.40 (0.42) | 0.45 (0.43) | |
|  | 4 | 0.59 (0.38) | 0.44 (0.40) | 0.44 (0.41) | 0.49 (0.50) | 0.47 (0.44) | 0.53 (0.45) | |
|  | 5 | 0.63 (0.38) | 0.48 (0.41) | 0.48 (0.41) | 0.51 (0.48) | 0.52 (0.43) | 0.57 (0.47) | |
|  | 6 | 0.65 (0.38) | 0.51 (0.41) | 0.51 (0.41) | 0.51 (0.45) | 0.55 (0.42) | 0.60 (0.42) | |
|  | 7 | 0.65 (0.36) | 0.53 (0.40) | 0.53 (0.41) | 0.50 (0.40) | 0.58 (0.42) | 0.64 (0.41) | |
| **Lipid (g/kg)** | 1 | 0.06 (0.13) | 0.02 (0.08) | 0.02 (0.08) | 0.10 (0.19) | 0.02 (0.10) | 0.02 (0.10) | |
|  | 2 | 0.03 (0.11) | 0.02 (0.10) | 0.02 (0.10) | 0.09 (0.19) | 0.02 (0.08) | 0.02 (0.08) | |
|  | 3 | 0.04 (0.13) | 0.03 (0.12) | 0.03 (0.11) | 0.09 (0.18) | 0.02 (0.09) | 0.02 (0.09) | |
|  | 4 | 0.05 (0.15) | 0.04 (0.13) | 0.04 (0.13) | 0.07 (0.16) | 0.03 (0.11) | 0.02 (0.10) | |
|  | 5 | 0.05 (0.16) | 0.05 (0.14) | 0.05 (0.14) | 0.07 (0.16) | 0.04 (0.12) | 0.03 (0.12) | |
|  | 6 | 0.06 (0.16) | 0.05 (0.15) | 0.05 (0.15) | 0.06 (0.15) | 0.05 (0.15) | 0.03 (0.12) | |
|  | 7 | 0.06 (0.17) | 0.06 (0.16) | 0.06 (0.16) | 0.05 (0.14) | 0.05 (0.16) | 0.03 (0.13) | |

^†^ Postoperative day 1 defined as the next day after surgery.

**Supplementary Table S7. Patients receiving prescribed parenteral energy and amino acid target doses^†^ on postoperative day^‡^** **7 among 19,145 adults in Japan who were fasting for 7 days or longer after gastroenterological cancer surgery from 2011 to 2022, by surgical site**

| **Components (*Target Doses*)** | **Esophagus** | **Stomach** | **Colon/**  **Rectum** | **Liver** | **Gallbladder/**  **Bile duct** | **Pancreas** |
| --- | --- | --- | --- | --- | --- | --- |
|  | N = 1,503 | N = 5,462 | N = 9,967 | N = 151 | N = 407 | N = 1,655 |
|  | n (%) | n (%) | n (%) | n (%) | n (%) | n (%) |
| **Energy**  *( ≥ 20 kcal/kg)* | 640 (42.6) | 1,189 (21.8) | 2,084 (20.9) | 32 (21.2) | 126 (31.0) | 467 (28.2) |
| **Amino acids**  *( ≥ 0.8 g/kg)* | 517 (34.4) | 1,530 (28.0) | 2,905 (29.1) | 34 (22.5) | 136 (33.4) | 626 (37.8) |

^†^ Target parenteral energy (≥ 20 kcal/kg) and amino acid (≥ 0.8 g/kg) doses based on guideline recommendatons^7^.

^‡^ Postoperative day 1 defined as the next day after surgery.
