# Supplementary material for: Nationwide medical database study for postoperative nutritional management in patients undergoing gastroenterological cancer surgery
Source: Ann Gastroenterol Surg. 2024 Nov 27;9(3):595–607. doi: 10.1002/ags3.12892 (PMC12080195; doi:10.1002/ags3.12892)
Supplement: Supplementary file 3 — Figure S1: Feeding routes during postoperative d 1–7 with/without laparoscopic surgery. [file AGS3-9-595-s002.pdf]

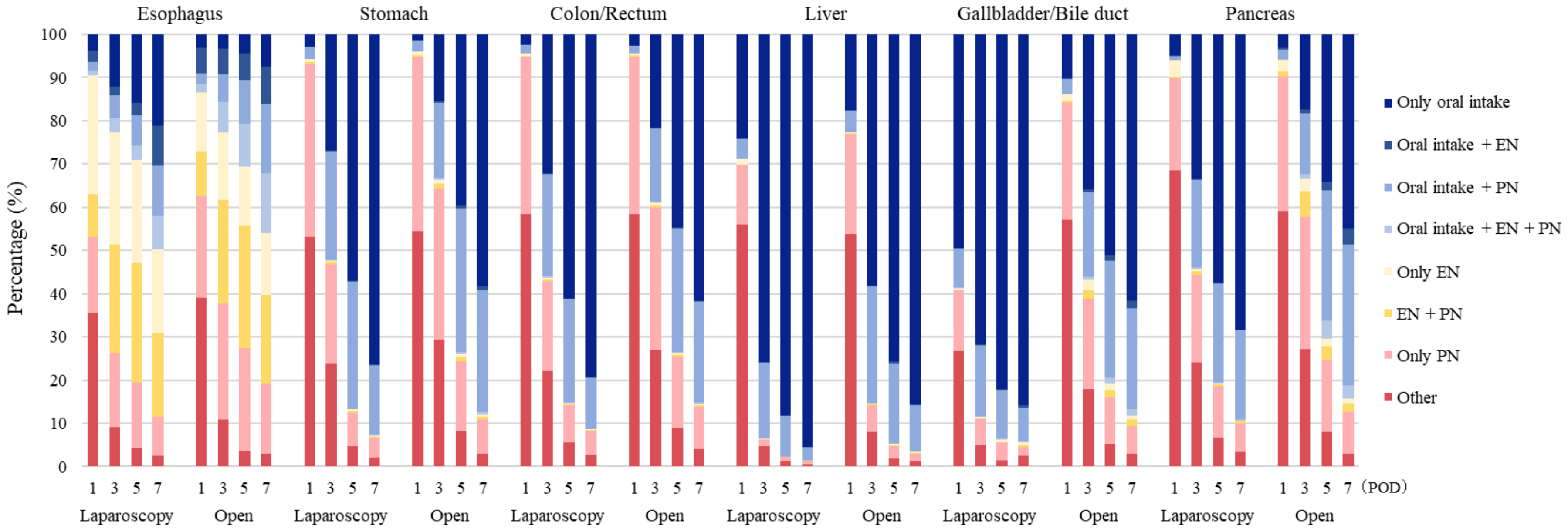

Supplementary Figure S1. Feeding routes[a] during postoperative days[b] 1–7 with/without laparoscopic surgery, received by 360,296 adult patients in Japan who underwent gastroenterological cancer surgery from 2011 to 2022, by surgical site[c].

[a] Oral intake defined as meals served; enteral nutrition (EN) defined as tube feedings prescribed; parenteral nutrition (PN) defined as intravenous solutions containing amino acids and/or lipid prescribed; and other defined as intravenous solutions containing only glucose and electrolytes prescribed.

[b] Postoperative day (POD) 1 defined as first day after surgery.

[c] Groups (with number of patients) based on surgical sites: esophagus (n = 14,784); stomach (n = 103,339); colon/rectum (n = 194,049); liver (n = 19,277); gallbladder/bile duct (n = 8,279); and pancreas (n = 20,568).
