# Supplementary material for: Nationwide medical database study for postoperative nutritional management in patients undergoing gastroenterological cancer surgery
Source: Ann Gastroenterol Surg. 2024 Nov 27;9(3):595–607. doi: 10.1002/ags3.12892 (PMC12080195; doi:10.1002/ags3.12892)
Supplement: Supplementary file 4 — Figure S2: Postoperative day of initiation of oral intake with/without postoperative complications. [file AGS3-9-595-s003.pdf]

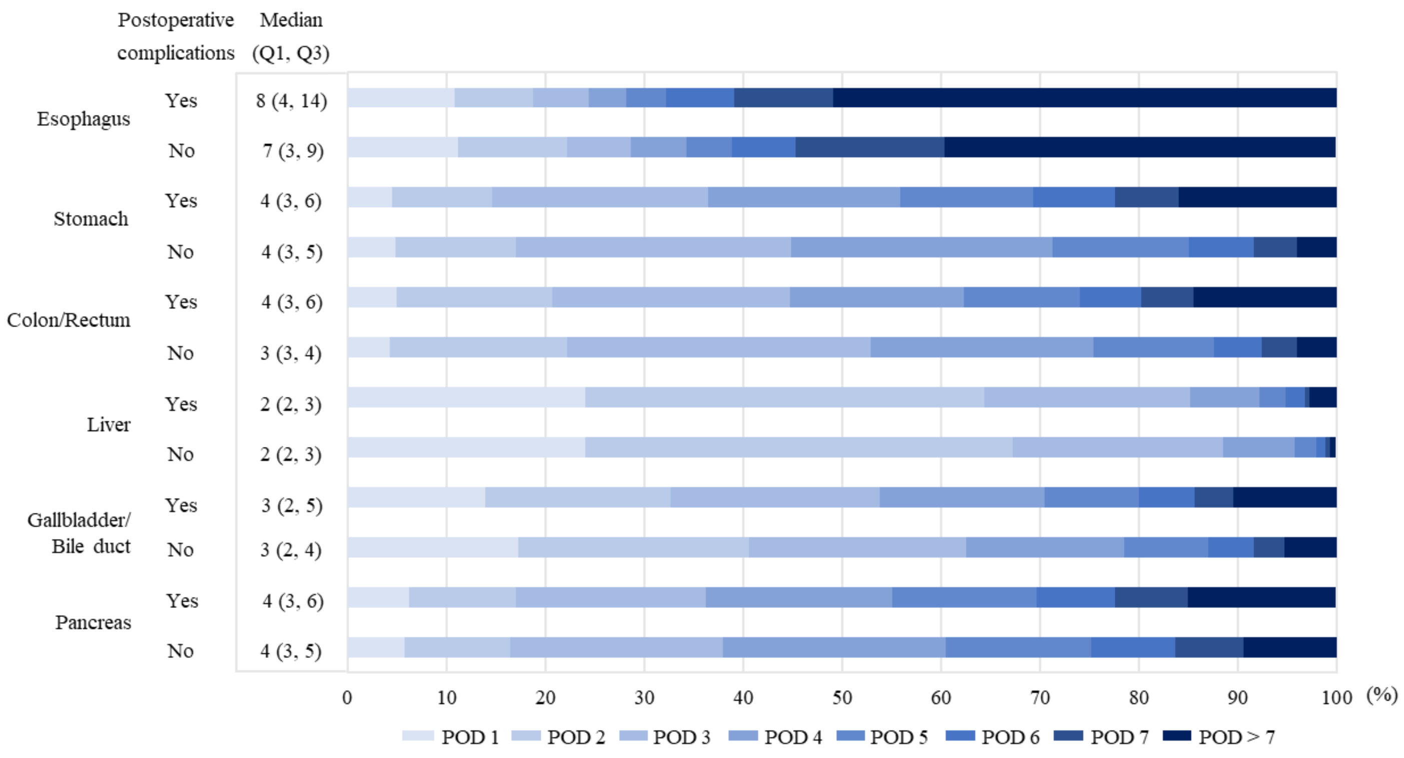

Supplementary Figure S2. Postoperative day[a] of initiation of oral intake with/without postoperative complications[b] among 359,138 adult patients in Japan who underwent gastroenterological cancer surgery and initiated oral intake during the hospitalized period from 2011 to 2022, by surgical site[c]. Median (quartile 1 [Q1], quartile [Q3]) day of initiation of oral intake within each patient group, and distribution within patient groups of days of initiation of oral intake, both by surgical site. Postoperative day of initiation of oral intake was recorded as ranging from postoperative day (POD) 1 to POD > 7.

[a] Postoperative day (POD) 1 defined as first day after surgery.

[b] Either of suture failure, wound infection, urinary tract infection, catheter infection, pneumonia.

[c] Groups (with numbers of patients) based on surgical sites: esophagus (n = 14,537); stomach (n = 103,052); colon/rectum (n = 193,532); liver (n = 19,257); gallbladder/bile duct (n = 8,229); and pancreas (n = 20,531).
